# HPV status determines prognostic gene expression methylation and immune infiltration in head and neck squamous cell carcinoma

**DOI:** 10.1007/s12672-026-04579-z

**Published:** 2026-03-03

**Authors:** Yifan Yang, Lingwa Wang, Ru Wang, Haiyang Li, Siyu Zhu, Jugao Fang, Ling Feng

**Affiliations:** 1 Department of Otorhinolaryngology, Head and Neck Surgery, Beijing Tongren Hospital, Capital Medical University, No.1 Dongjiaominxiang Street, Beijing, 100730 China; 2 Key Laboratory of Otorhinolaryngology, Head and Neck Surgery, Beijing Institute of Otorhinolaryngology, Beijing, China; 3 Department of Otorhinolaryngology, Head and Neck Surgery, Beijing Shijitan Hospital, Capital Medical University, No.10 Tieyi Road, Beijing, 100038 China

**Keywords:** Head and neck squamous cell carcinoma, HPV, DNA methylation, Tumor immune microenvironment, Database

## Abstract

**Purpose:**

Head and neck squamous cell carcinoma (HNSCC) continues to be a deadly cancer with heterogeneous molecular characteristics and poor survival outcomes, particularly in HPV- patients. This study aimed to identify key overexpressed genes that drive HNSCC progression, evaluate their prognostic value, and explore associations with HPV status, promoter methylation, and changes in the immune microenvironment.

**Methods:**

Using bioinformatics tools (UALCAN, HPA, TISIDB), we analyzed TCGA-HNSC data to evaluate gene expression, survival correlations, HPV subgroup differences, promoter methylation, and immune infiltration patterns. Statistical significance was defined as *p* < 0.05.

**Results:**

Among the top 50 overexpressed genes in HNSCC, eight (LAMC2, CDKN2A, MFAP2, CTHRC1, CXCL13, FST, SPP1, PLAU) exhibited significant survival associations (*p* < 0.05). HPV- tumors demonstrated marked upregulation of LAMC2, CTHRC1, FST, SPP1, and PLAU, alongside downregulation of CDKN2A and CXCL13. Promoter hypomethylation in tumor tissues correlated with overexpression of LAMC2, CTHRC1, CXCL13, FST, SPP1, and PLAU, whereas CDKN2A showed hypermethylation. Immune infiltration analysis revealed strong correlations between these genes and immunosuppressive Tregs or cytotoxic T-cell depletion.

**Conclusion:**

This study identifies eight prognostic biomarkers in HNSCC linked to HPV-driven heterogeneity. These genes could be potential targets for therapy in combination with immunotherapy and epigenetic regulators, helping to overcome tumor resistance in HNSCC with different HPV status.

**Supplementary Information:**

The online version contains supplementary material available at 10.1007/s12672-026-04579-z.

## Introduction

Head and neck squamous cell carcinoma (HNSCC) ranks as the sixth most common malignancy globally, with approximately 890,000 new cases and resulting in 450,000 deaths annually [1]. HNSCC is strongly associated with risk factors such as smoking, alcohol consumption, and human papillomavirus (HPV) infection. HPV + and HPV− HNSCC demonstrate different molecular characteristics and clinical outcomes. HPV+ tumors, which are most commonly found in the oropharynx, tend to be less aggressive, whereas HPV− tumors are often driven by TP53 mutations and chromosomal instability, resulting in a poorer prognosis [2, 3]. Treatment strategies for HNSCC depend on the disease stage. Surgical intervention remains a curative for early-stage disease, but over 50% of patients are diagnosed with locally advanced disease, which typically requires comprehensive therapy involving radiotherapy, chemotherapy, or targeted therapy [4, 5]. While concurrent chemoradiation therapy enhances 5-year survival by approximately 6.5%, it is often associated with notable side effects and toxicity [6]. Epidermal growth factor receptor (EGFR) inhibitors, such as cetuximab, have demonstrated modest survival benefits in patients with recurrent or metastatic, though the therapeutic efficacy remains limited [7]. Recently, immune checkpoint inhibitors (ICIs) such as pembrolizumab and nivolumab have revolutionized the treatment of recurrent or metastatic HNSCC. The KEYNOTE-048 trial showed that pembrolizumab monotherapy or in combination with chemotherapy led to improved overall survival (OS) in PD-L1–high patients compared to conventional chemotherapy [8]. Neoadjuvant immunotherapy has demonstrated potential in pathologic response rates and lowering the risk of recurrence [9]. However, several challenges remain, including limited response rate to immunotherapy (approximately 20–30%), failure to enhance survival outcomes in Phase III trials involving dual immune checkpoint blockade (such as PD-1 and CTLA-4 inhibitors), and differences in the immune microenvironment heterogeneity between HPV + and HPV− tumors [10]. HPV− tumors are distinguished by an abundance of regulatory T cells (Tregs), myeloid-derived suppressor cells (MDSCs), and upregulated of immune checkpoint molecules, which collectively facilitate immune evasion [11]. 

Molecular and epigenetic differences contribute to the heterogeneity of HNSCC. HPV+ tumors are characterized by abnormal promoter methylation of host genes, such as CDKN2A silencing, which is associated with cell cycle regulation. In contrast, HPV− tumors exhibit global hypomethylation that activates oncogenic signaling pathways like PI3K/AKT [12]. The tumor immune microenvironment (TME) in HPV+ HNSCC is marked by high immune cell infiltration, including cytotoxic T lymphocytes (CTLs) and tertiary lymphoid structures, which may enhance responsiveness to ICIs [13]. In contrast, HPV− tumors display an immunosuppressive TME, contributing to resistance against immunotherapy [14]. In HNSCC, several biomarkers have emerged, showing promising results in diagnosis, early detection and prognosis of HNSCC. HPV DNA/p16 for the determination of HPV status, PET imaging and PD-L1 are validated diagnostic and prognostic/predictive biomarkers currently used in clinical practice. A multitude of molecular alterations from gene mutations and telomerase activity to chemokine receptors and liquid biopsy markers have been associated with prognosis in HNSCC [15, 16]. However, integrating these disparate factors into a coherent understanding of tumor biology, particularly as influenced by HPV status, remains a critical challenge for improving patient stratification. Future research priorities in HNSCC treatment involve investing new therapeutic targets, understanding why some patients respond and some are resistant to these treatments, and designing combination therapies such as paring immunotherapy with radiotherapy or targeted therapies to against treatment resistance. Advances in non-invasive screening methods based on methylation biomarkers may enhance early diagnosis. Integrating multi-omics data to design precision therapies to specific subtypes will be critical for advancing personalized treatment and improving patient outcomes. Additionally, HPV status continues to be a key factor of heterogeneity in HNSCC, and understanding its molecular mechanisms could find more effective treatment strategies. Thus, this study aimed to identify key overexpressed genes driving HNSCCC progression, assess their prognostic value, and explore associations with HPV status, promoter methylation, and immune microenvironment dynamics.

## Methods

### Data sources and analytical work flow

In this study, comprehensive multi-omics and clinical data were integrated from publicly accessible databases to enable analysis. The primary molecular profiling data and detailed clinical information were obtained from The Cancer Genome Atlas (TCGA) HNSC cohort through the UALCAN platform (University of Alabama Cancer Analysis Portal) (http://ualcan.path.uab.edu/index.html), including RNA-sequencing profiles normalized using fragments per kilobase of transcript per million fragments mapped (FPKM) methodology, this cohort included treatment-naïve primary tumor specimens, with molecular subtyping revealing 434 HPV- patients and 80 HPV+ patients, along with 44 normal mucosal samples serving as controls. The top 50 overexpressed genes in HNSCC verse normal tissues were identified using UALCAN’s expression-TCGA module (threshold: |log2FC| > 2, *p* < 0.01). Differential expression between HPV- and HPV+ tumors was assessed via UALCAN’s subgroup analysis (Student’s t-test, *p* < 0.05). To independently validate the expression patterns of the candidate genes identified from the TCGA analysis, we utilized the GENT2 database (http://gent2.appex.kr/gent2/), which provides integrated mRNA expression profiles compiled from multiple independent datasets in the Gene Expression Omnibus (GEO).

DNA methylation data generated through the Illumina Infinium HumanMethylation450 BeadChip platform, this cohort included 528 treatment-naïve primary tumor specimens, along with 50 normal mucosal samples serving as controls. Methylation β-values (ranging 0–1) for gene promoters (TSS200 regions) were compared between tumors and normal tissues (Mann-Whitney U test, *p* < 0.05). We extended our methylation analysis to include CpG island shores, gene body regions, and enhancers using UCSC Genome Browser (https://genome.ucsc.edu/index.html) and THInCR databases (https://mymryklab.ca/THInCR/thincr-methyl-all).

To validate protein-level expression patterns corresponding to transcriptomic findings, immunohistochemistry (IHC) was obtained from the Human Protein Atlas (HPA) database (www.proteinatlas.org).

To assess TME dynamics, we analyzed immune cell infiltration levels (including CD4 + T lymphocytes, CD8 + cytotoxic T cells, and regulatory T cells (Tregs)) with gene expression correlations using the Tumor-Immune System Interactions Database (TISIDB). (http://cis.hku.hk/TISIDB). Spearman’s rank correlation coefficients (ρ) between gene expression and immune cell abundance (CIBERSORT-estimated) were computed using TISIDB (*p* < 0.05).

This integrated analysis allowed for a comprehensive cross-platform validation that included transcriptional regulation, epigenetic modifications, proteomic analysis, and immune context assessment to elucidate molecular mechanisms in HNSCC.

### Statistical analysis

All statistical analyses were performed using UALCAN and TISIDB’s built-in tools. Survival hazard ratios (HRs) and 95% confidence intervals (CIs) were calculated via Cox proportional hazards models.

## Results

### Prognostic overexpressed genes in HNSCC

**Fig. 1:**
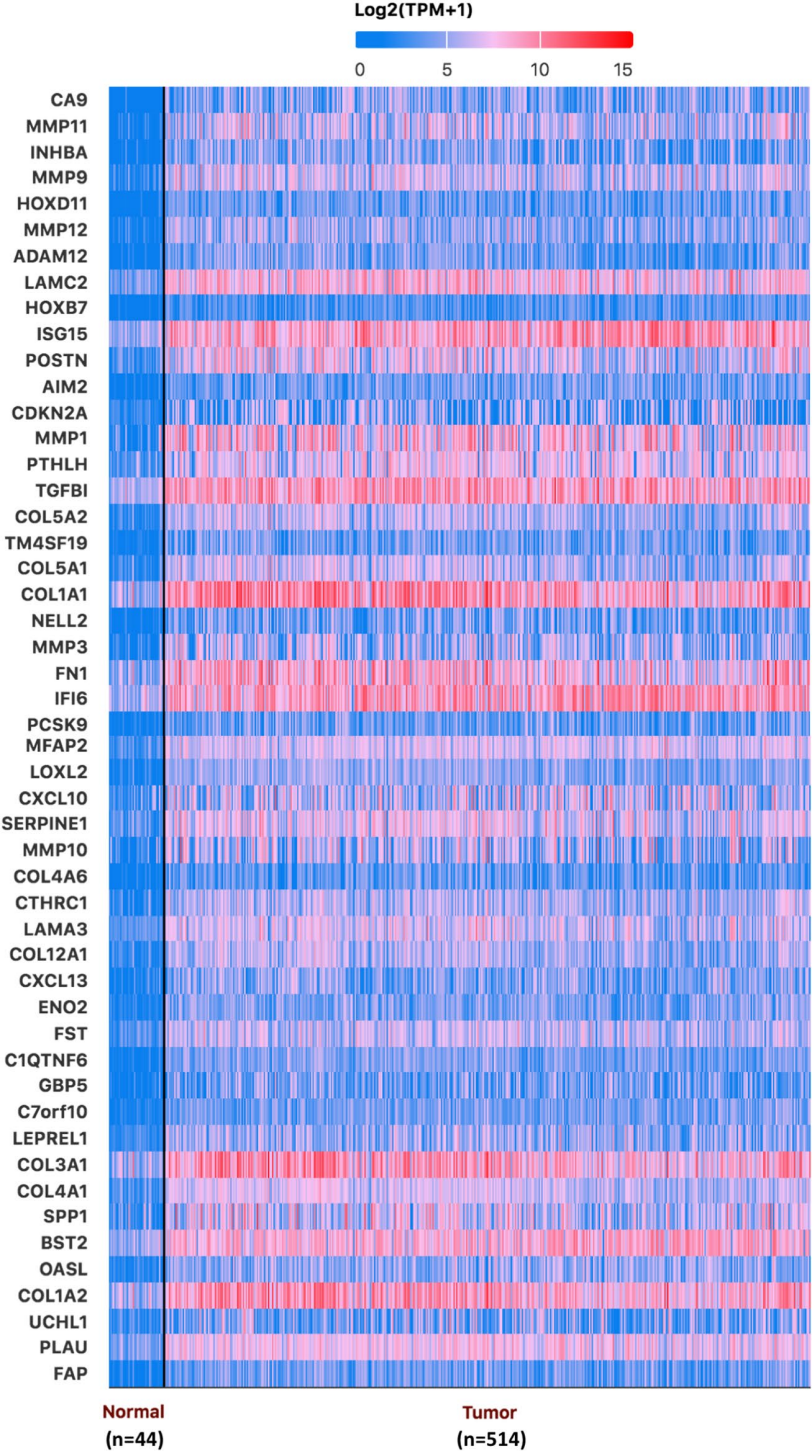
Differential gene expression profiling in HNSCC. Heatmap visualization of 50 significantly overexpressed genes (log2[TPM + 1] transformation) through UALCAN analysis, comparing primary HNSCC tumors (*n* = 514) with normal controls (*n* = 44). Color gradient represents normalized expression levels (blue: low expression; red: high expression)

**Fig. 2:**
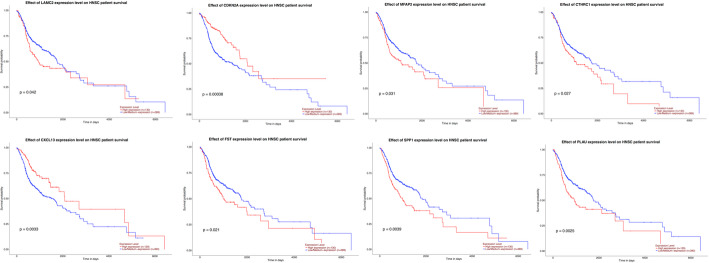
Prognostic significance of candidate genes in HNSCC survival outcomes. Multivariate Cox regression analysis found overall survival associations in eight genes (LAMC2, CDKN2A, MFAP2, CTHRC1, CXCL13, FST, SPP1, PLAU) using TCGA-HNSC cohort data via UALCAN platform. Kaplan-Meier curves show significant survival stratification between high (upper quartile) and low (lower quartile) expression groups

**Fig. 3:**
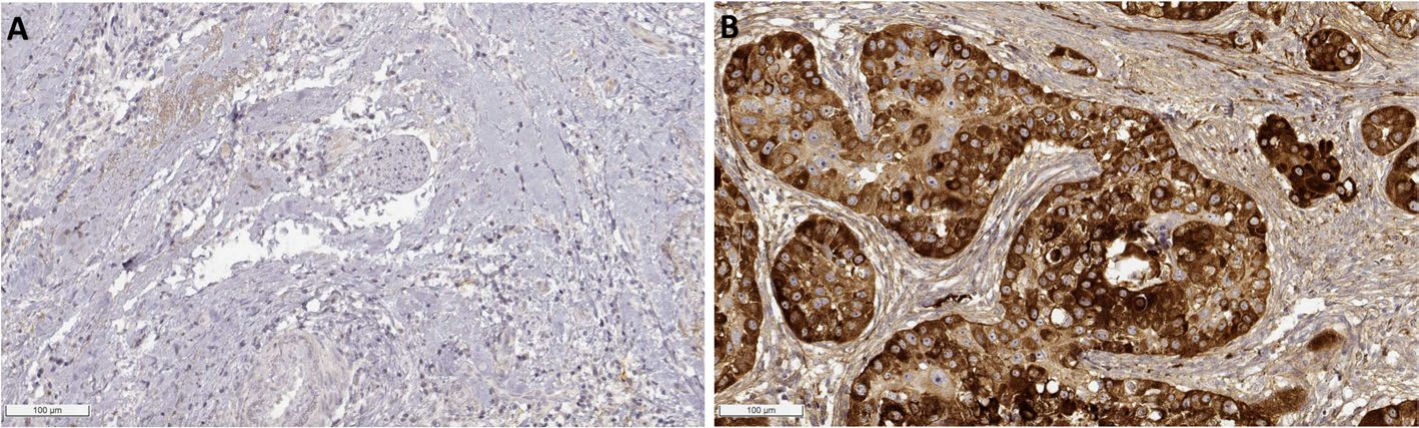
Protein-level validation of LAMC2 overexpression in HNSCC. Immunohistochemical staining from HPA: (**A**) Absent LAMC2 immunoreactivity in normal mucosa; (**B**) Strong LAMC2 expression in HNSCC tissue

UALCAN analysis revealed 50 genes significantly overexpressed in HNSCC (Fig. 1), with eight genes demonstrating intense survival associations: LAMC2, CDKN2A, MFAP2, CTHRC1, CXCL13, FST, SPP1, and PLAU (*p* < 0.05) (Fig. 2). We validated the expression patterns of the eight genes using the integrated GEO database. Consistent with our primary analysis, seven genes (LAMC2, MFAP2, CTHRC1, CXCL13, FST, SPP1, and PLAU) were significantly overexpressed in head and neck cancer tissues compared to normal controls (all *p* < 0.01), while CDKN2A did not reach statistical significance in this analysis (Supplementary Figure S1). Notably, elevated expression of LAMC2 (*p* = 0.042), MFAP2 (*p* = 0.031), CTHRC1 (*p* = 0.027), FST (*p* = 0.021), SPP1 (*p* = 0.0039), and PLAU (*p* = 0.0025) correlated with poor overall survival, identifying them as high-risk markers. Conversely, reduced CDKN2A (*p* = 0.00038) and CXCL13 (*p* = 0.0033) expression levels predicted poor survival outcomes, suggesting their potential protective roles in tumor progression. Given the distinct molecular landscapes of HPV + and HPV- HNSCC, we performed subgroup survival analyses to evaluate whether the prognostic associations of the eight candidate genes were dependent on HPV status. Meaningful analysis was not feasible in the HPV+ cohort due to a low number of survival events (*n* = 1). Within the HPV- cohort, high expression of PLAU (*p* = 0.03) and MFAP2 (*p* = 0.02) remained significantly associated with inferior overall survival (Supplementary Figure S2). These findings suggest that the prognostic impact of PLAU and MFAP2 is particularly relevant in HPV- tumors. Moreover, pathology section by HPA Immunohistochemical database by revealed that LAMC2 protein expression was much higher in HNSCC tissues (CAB078165) than in normal control tissues (CAB004257) (Fig. 3).

### HPV status modulates gene expression

**Fig. 4:**
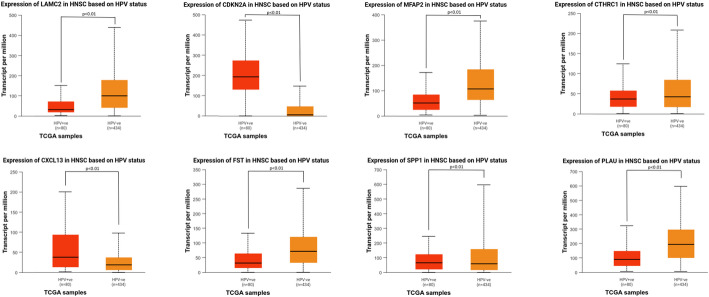
HPV status-dependent transcriptional heterogeneity. Comparative transcriptome analysis of HPV+ versus HPV- HNSCC tumors identifies eight differentially expressed genes among top 50 overexpressed candidates. Boxplots display median expression values with interquartile ranges (UALCAN; TPM normalization, HPV+: red; HPV-: orange)

Among the top 50 overexpressed genes in HNSCC, eight showed significant differential expression between HPV + and HPV- tumors. HPV- tumors exhibited significant upregulation of oncogenic drivers including LAMC2, MFAP2, CTHRC1, FST, SPP1, and PLAU (*p* < 0.01), accompanied by marked downregulation of CDKN2A and CXCL13 (*p* < 0.01) (Fig. 4). This bidirectional dysregulation pattern was consistent in trend with the high-risk and protective prognostic markers identified in survival analyses.

### Methylation alterations

**Fig. 5:**
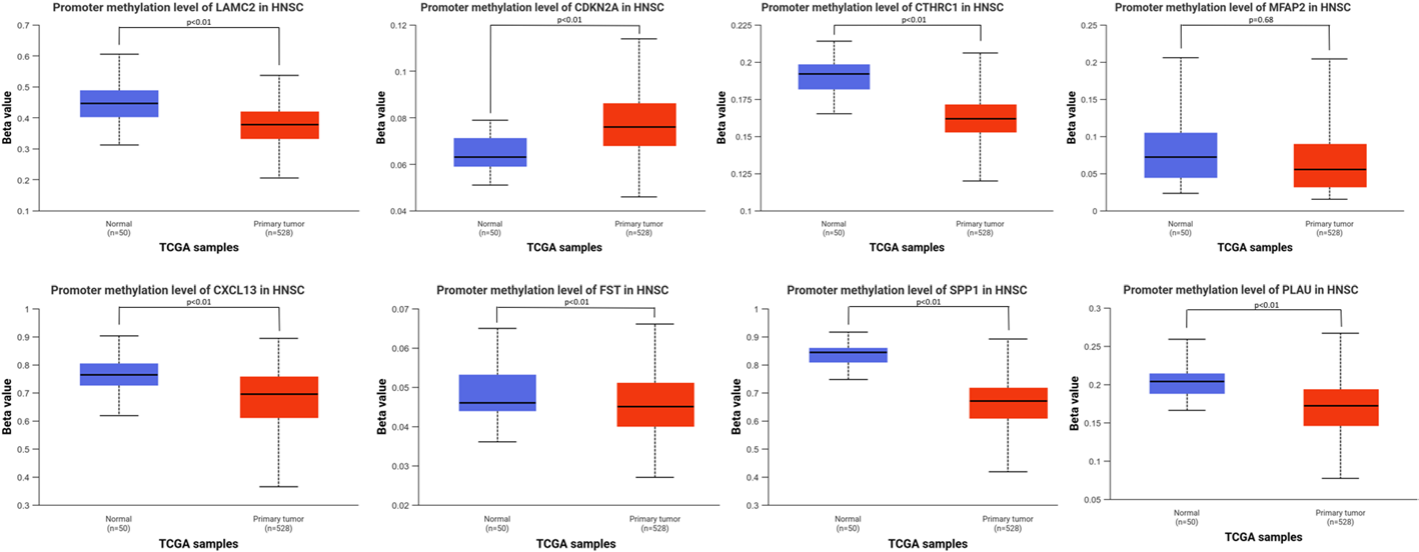
Epigenetic dysregulation in HNSCC carcinogenesis. Genome-wide methylation analysis (Illumina Infinium HumanMethylation450 BeadChip) reveals significant tumor-specific tumor-specific methylation within promoter-associated CpG islands. Representative shown with β-value distributions (normal: blue; tumor: red)

Methylation analysis demonstrated tumor-specific epigenetic dysregulation in HNSCC (Fig. 5), with pronounced promoter hypomethylation observed in key genes including LAMC2, CTHRC1, CXCL13, FST, SPP1, PLAU (p all < 0.01). Conversely, CDKN2A exhibited significant hypermethylation (*p* < 0.01). We extended our methylation analysis to include CpG island shores, gene body regions. We identified distinct methylation patterns across these regions for the eight prognostic genes (Table 1, Figure S3). Notably, while promoter hypomethylation of LAMC2, CTHRC1, FST, SPP1, and PLAU correlated with their overexpression in HPV- HNSCCs, gene body hypermethylation was observed in LAMC2 and CTHRC1, suggesting complex transcriptional regulation beyond promoter activity. Conversely, CDKN2A exhibited consistent hypermethylation across promoter, gene body, and CpG island regions, reinforcing its silencing in HPV- tumors. Enhancer methylation data were limited due to probe availability, yet our findings underscore the multi-layered epigenetic dysregulation in HNSCC, which may further explain HPV-driven transcriptional heterogeneity.

**Table 1:** Comparative methylation profiles and expression patterns of eight prognostic genes in HPV- versus HPV+ HNSCC

Gene	Genomic Feature	Probe ID	Differential Methylation (HPV- vs. HPV+)	mRNA Expression (HPV- vs. HPV+)
LAMC2	Promoter	cg10519299	↓ Hypomethylated	↑ Upregulated
	Gene Body	cg23696949	↑ Hypermethylated	
	Gene Body	cg01417625	↓ Hypomethylated	
MFAP2	Promoter (CpG Island)	cg05985737	↑ Hypermethylated	↑ Upregulated
	Gene Body	cg05055766	↓ Hypomethylated	
	Gene Body	cg21304454	↑ Hypermethylated	
CTHRC1	Promoter (CpG Island)	cg21936543	↑ Hypermethylated	↑ Upregulated
	Gene Body	cg18919478	↓ Hypomethylated	
	Gene Body	cg19188612	↑ Hypermethylated	
FST	Promoter (CpG Island)	cg25645657	↓ Hypomethylated	↑ Upregulated
	Gene Body	cg05636869	↓ Hypomethylated	
SPP1	Promoter	cg15460348	↓ Hypomethylated	↑ Upregulated
	Gene Body	cg11226901	↓ Hypomethylated	
PLAU	Promoter (CpG Island)	cg26457761	↓ Hypomethylated	↑ Upregulated
	Gene Body	cg04939496	↓ Hypomethylated	
CDKN2A	Promoter (CpG Island)	cg10848754	↑ Hypermethylated	↓ Downregulated
	Gene Body	cg13601799	↑ Hypermethylated	
CXCL13	Gene Body	cg06662476	↓ Hypomethylated	↓ Downregulated

For each gene, representative methylation probes in promoter and gene body regions are shown. Arrows indicate the direction of change in HPV- tumors relative to HPV+ tumors (↑: increase; ↓: decrease). Significant hypomethylation or hypermethylation are derived from Supplementary Fig. 1 (all *P* < 0.05). Corresponding mRNA expression changes (upregulated or downregulated in HPV- tumors) are derived from Fig. 4 (all *P* < 0.01)

### Immune microenvironment associations

**Fig. 6:**
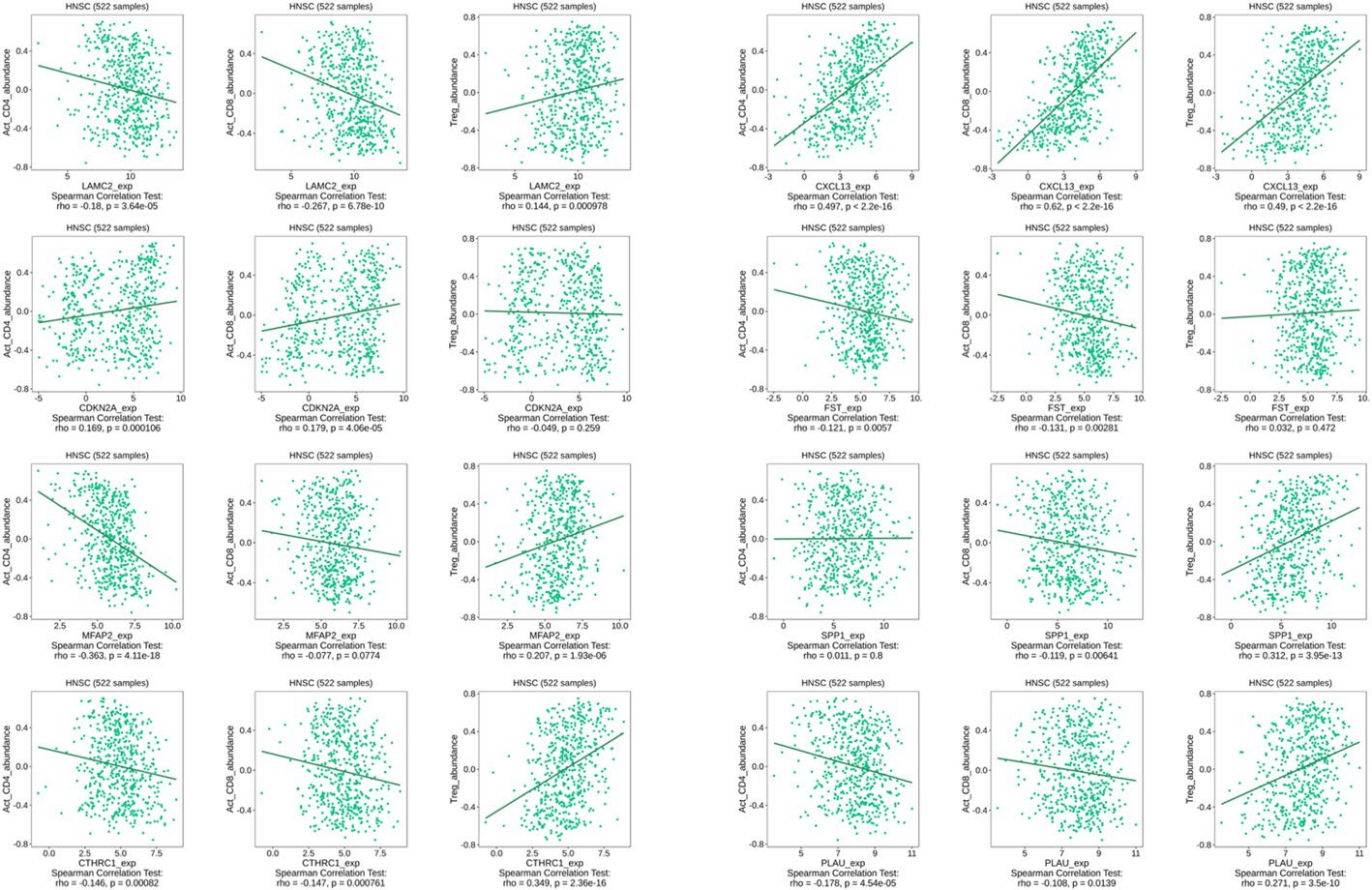
Immune microenvironment interactions of candidate genes TISIDB database analysis of tumor-immune system interactions (CD4 + T cells, CD8 + T cells, Tregs) using Spearman’s correlation

Analysis of immune cell co-expression relationships using the TISIDB database revealed distinct gene-specific patterns (Fig. 6). LAMC2 exhibited significant negative correlations with CD4⁺T cells (*p* < 0.01) and CD8⁺T cells (*p* < 0.01), alongside a positive correlation with Treg cells (*p* < 0.01). CDKN2A showed positive associations with CD4⁺T and CD8⁺T cells (*p* < 0.01), but its negative correlation with Treg cells lacked statistical significance. For MFAP2, a positive correlation with Treg cells (*p* < 0.01) and a negative trend with CD4⁺T cells (*p* < 0.01) were observed, but its negative correlation with CD8⁺T cells lacked statistical. CTHRC1 displayed negative correlations with both CD4⁺T and CD8⁺T cells (*p* < 0.01) and a positive correlation with Treg cells (*p* < 0.01). CXCL13 demonstrated consistently positive associations across CD4⁺T, CD8⁺T cells, and Treg cells (*p* < 0.01). FST correlated negatively with CD4⁺T and CD8⁺T cells (*p* < 0.01), while its positive association with Tregs remained non-significant. SPP1 showed a significant negative correlation with CD8⁺T cells (*p* < 0.01) and a positive correlation with Tregs (*p* < 0.01), though its association with CD4⁺T cells was non-significant. PLAU exhibited consistent negative correlations with CD4⁺T (*p* < 0.01) and CD8⁺T cells (*p* < 0.05) and a positive correlation with Tregs (*p* < 0.01). These findings collectively suggest tumor microenvironment remodeling through gene-specific modulation of cytotoxic and regulatory T cell populations.

## Discussion

In this study, LAMC2, CDKN2A, MFAP2, CTHRC1, CXCL13, FST, SPP1, and PLAU were identified as key molecular drivers of HNSCC progression highlights the complex interactions between genetic, epigenetic, and immune microenvironmental factors that shape tumor behavior. These genes show distinct expression patterns associated with HPV status, promoter methylation alterations, and immune evasion mechanisms. This demonstrates their potential as prognostic biomarkers and therapeutic targets. Our HPV-stratified survival analysis further clarifies the context-dependent prognostic value of the identified genes. The significant association of MFAP2 and PLAU with poor survival specifically in the HPV- cohort underscores their role as potential biomarkers for this aggressive subtype. The lack of significant associations for the other six genes in this subgroup may reflect the distinct oncogenic drivers and immune microenvironment of HPV- tumors, where factors such as TP53 mutation and widespread hypomethylation might dominate the prognostic landscape. These results emphasize the necessity of considering HPV status when evaluating prognostic biomarkers in HNSCC, as their clinical utility may be confined to specific etiological contexts. The TCGA-HNSC cohort reflects the established epidemiological link between HPV positivity and oropharyngeal location. Consequently, our findings regarding HPV+ tumors most directly apply to oropharyngeal squamous cell carcinoma (OPSCC). For HPV- tumors, which include multiple subsites (primarily oral cavity and larynx), the identified biomarkers likely reflect shared high-risk biology across non-oropharyngeal diseases. While anatomical context remains relevant, our study underscores that HPV status is a dominant molecular stratify, delineating distinct prognostic and immune profiles in HNSCC. Further subsite-stratified studies may refine these associations.

The significant upregulation of LAMC2, CTHRC1, and PLAU in HPV- HNSCC is consistent with their established roles in extracellular matrix remodeling, epithelial-mesenchymal transition (EMT), and metastasis. As a laminin subunit critical for basement membrane integrity, LAMC2 promotes tumor cell migration and invasion by activating integrin-mediated signaling pathways [17]. Its overexpression in HPV- HNSCCs correlates with promoter region hypomethylation, suggesting that epigenetic suppression is a key driver of its oncogenic activity. Similarly, CTHRC1, a secreted glycoprotein involved in collagen deposition and Wnt signaling, promotes tumor cell motility and angiogenesis [18]. Hypomethylation-driven overexpression of CTHRC1 may account for the aggressive stromal remodeling features of HPV- HNSCCs, which are often characterized by a tumor microenvironment that is resistant to conventional therapies [19]. PLAU (urokinase-type plasminogen activator) is another example of the integration of proteolytic activity and immunomodulation. Its overexpression not only enhances matrix degradation but also polarizes macrophages to the tumor-associated M2 phenotype [20].

CDKN2A encoded p16INK4a and p14ARF, both are key tumor suppressors regulating the Rb and p53 pathways [21]. In HPV- HNSCCs, the inverse association between CDKN2A hypermethylation and transcriptional silencing strengthened the role of epigenetic dysregulation in cell cycle control [22]. In HPV+ tumors, E7-mediated Rb degradation often causes p16 overexpression, while HPV− tumors rely on CDKN2A promoter hypermethylation to bypass cell cycle checkpoints [23]. This highlights the need to stratify HNSCC by HPV status when evaluating CDKN2A as a therapeutic target, as reactivation strategies like demethylating agents may only benefit the HPV- subgroup. These epigenetic alterations can serve as non-invasive biomarkers for early detection or monitoring therapy response. For instance, circulating tumor DNA with CDKN2A hypermethylation has been suggested as a liquid biopsy target for HPV- HNSCC [24].

CXCL13, a chemokine linked to lymphangiogenesis, is down-regulated in HPV- tumors. This may explain the scarcity of tertiary lymphoid structures in these immunologically “cold” microenvironments [25]. The correlation between CXCL13 downregulation and cytotoxic T-cell exhaustion implies that restoring its expression could enhance antitumor immunity, particularly when combined with immune checkpoint inhibitors [26].

LAMC2, CTHRC1, and PLAU are consistently linked to Treg infiltration and cytotoxic T cell exclusion, reflecting immune evasion in HPV− tumors. Tregs, known to suppress effector T cell responses via cytokine secretion such as IL-10, TGF-β, and metabolic competition, may be recruited by tumor-associated fibroblasts expressing these genes through chemotactic signals [27]. Conversely, CDKN2A and CXCL13 are positively related to CD8 + T-cell infiltration, suggesting that their absence in HPV− tumors creates an immunological niche for tumor progression. These findings align with recent research indicating that HPV− HNSCC is associated with higher levels of PD-1 + CD8+ T cell exhaustion and fewer dendritic cell subsets capable of initiating antitumor responses [28]. It is important to note that our immune infiltration analysis relies on computational deconvolution, which provides inferred rather than direct quantification of immune cell abundances. Future validation using spatial profiling techniques on independent cohorts would be valuable to confirm these interactions within the tissue architecture. More critically, to bridge the in-silico findings with clinical practice, it is essential to consider how the immune landscape defined by our eight-gene signature relates to established biomarkers for immunotherapy, particularly PD-L1 expression.

MFAP2, a microfibril-associated protein, is significantly overexpressed in HNSCC, and is linked to aggressive clinical features like tumor size, lymph node metastasis, and advanced TNM stage [29]. Mechanistically, it is a role in initiating extracellular matrix remodeling and activation, potentially promoting local invasion before metastasis. Our analysis reveals a complex methylation landscape for MFAP2 in HPV- tumors, characterized by promoter/CpG island hypermethylation and mixed (hyper/hypo) gene body methylation, indicating regulation beyond canonical epigenetic silencing. Specifically, hypomethylation of putative enhancer regions near MFAP2 could increase its accessibility to transcription factors, a mechanism observed in other tobacco-related cancers [30]. Additionally, constitutive Wnt/β-catenin or Notch1 signaling may directly enhance MFAP2 transcription [29, 31], while loss of tumor-suppressive miRNAs could further elevate its expression [32]. Thus, MFAP2 upregulation likely results from enhancer remodeling, transcription factor activation, and miRNA dysregulation, highlighting a complex regulatory network beyond promoter methylation.

FST, a TGF-β superfamily antagonist, is overexpressed in HNSCC cell lines and patient tissues, promoting tumor proliferation, invasion, and migration. Gene knockout shows FST silencing reduces clonogenicity, Transwell migration, and xenograft growth, and increases apoptosis via caspase-3. Transcriptomic analysis indicates FST depletion suppresses TGF-β pathway, suggesting FST may counteract TGF-β’s tumor-suppressive effects and later enhance its pro-metastatic signaling [33]. Elevated FST in HPV- tumors highlight its role in driving aggressive phenotype via cancer-associated fibroblast-mediated stromal cooperation. Targeting FST or its downstream effectors like activin A may disrupt TGF-β-mediated immune evasion and stromal decalcification [34].

SPP1, mainly expressed in tumor associated macrophages (TAMs), is a key tumor-immune interaction mediator [35]. SPP1 + macrophages are enriched in HNSCC tumors, linked to poor prognosis and an immunosuppressive microenvironment. These macrophages secrete TNF-α and IL−1β via NF-κB activation, promoting tumor cell proliferation and migration, while recruiting Tregs and inhibiting cytotoxic CD8 + T cell infiltration. Preclinical studies indicate that blocking SPP1 + macrophages or neutralizing TNF-α/IL − 1β reduces tumor. SPP1 acts as both a secreted cytokine OPN and a macrophage marker, serving as a prognostic biomarker and therapeutic target [36, 37]. Depleting SPP1 + macrophages may reverse the immunosuppressive niche in HPV- HNSCC [38].

The current clinical decision-making for immune checkpoint inhibitor therapy in HNSCC heavily relies on PD-L1 expression (Combined Positive Score, CPS) [39]. Our study suggests that the eight-gene prognostic signature may offer complementary information. While PD-L1 primarily reflects one mechanism of T-cell exhaustion, our signature encompasses broader tumor microenvironmental features: LAMC2 and CTHRC1 are linked to fibrotic, T-cell-excluded niches; SPP1 marks immunosuppressive macrophages; and CXCL13 is associated with tertiary lymphoid structures. A tumor might be PD-L1 positive yet harbor a high-risk signature indicative of stromal barrier and innate immune suppression, which could contribute to primary resistance to anti-PD-1 monotherapy. Therefore, combining PD-L1 status with a signature like ours might improve patient stratification, identifying those with PD-L1 + cold tumors who could be candidates for combination therapies targeting the extracellular matrix (LAMC2/CTHRC1) or tumor-associated macrophages (SPP1). Future prospective studies are warranted to evaluate the additive predictive value of this multi-gene signature alongside PD-L1 in patients receiving immunotherapy.

Despite these advances, several limitations must be acknowledged. Our findings, derived from public datasets, await experimental validation. Gain- and loss-of-function studies are planned to definitively assess the functional contribution of 8-genes to tumor progression. Functional validation of identified genes using in vitro or in vivo models is needed to confirm their roles in immune modulation and metastasis. Future research should integrate single-cell sequencing to analyze cell-type-specific expression and spatial interactions in the tumor microenvironment.

## Conclusion

This study identifies LAMC2, CDKN2A, MFAP2, CTHRC1, CXCL13, FST, SPP1, and PLAU as key prognostic biomarkers for HNSCC, highlighting the molecular and immune heterogeneity driven by HPV. These genes could be compelling targets for therapy in combination with immunotherapy and epigenetic regulators, potentially helping to overcome tumor resistance in HNSCC with different HPV status. In the future, the integration of multi-omics and non-invasive biomarkers will advance personalized treatment approaches.

## Supplementary Information

Supplementary Material 1 (Figure S1. Validation of candidate gene expression in HNSCC using the GENT2 database. Box plots compare the mRNA expression levels of the eight prognostic genes between Head and neck cancer (red) and Head and neck Normal (blue) tissue samples. Y-axis represents log2-transformed normalized expression values. Seven genes (LAMC2, MFAP2, CTHRC1, CXCL13, FST, SPP1, and PLAU) were significantly overexpressed in tumors (P all<0.01). CDKN2A showed a non-significant trend in this aggregated analysis.).

Supplementary Material 2 (Figure S2. HPV-stratified survival analysis of candidate genes in the TCGA-HNSC cohort. Kaplan-Meier curves depict overall survival in the HPV- subgroup stratified by high vs. low expression (median cut-off) of LAMC2, MFAP2, CTHRC1, FST, SPP1, PLAU, CDKN2A, and CXCL13. Log-rank p-values are indicated. Analysis of the HPV+ subgroup was not feasible due to an insufficient number of survival events.).

Supplementary Material 3 (Figure S3. Multi-region methylation analysis of eight prognostic genes in HPV- versus HPV+ HNSCC. Comparative methylation profiles across genomic features. Bar graphs display the methylation β-values for representative probes of each gene in HPV- tumors versus HPV+ tumors. Probes are grouped by genomic feature: Promoter, Gene Body, and CpG islands. Only features with available probe data are shown.).

## Data Availability

All data generated or analyzed during this study are included in this published article. The original data are publicly available from The Cancer Genome Atlas (TCGA) Head-Neck Squamous Cell Carcinoma (HNSC) cohort (https://portal.gdc.cancer.gov/projects/TCGA-HNSC) and were analyzed using the UALCAN, Human Protein Atlas, and TISIDB platforms as described in the Methods section.
