# Supplementary material for: HPV status determines prognostic gene expression methylation and immune infiltration in head and neck squamous cell carcinoma
Source: Discov Oncol. 2026 Mar 3;17:553. doi: 10.1007/s12672-026-04579-z (PMC13065962; doi:10.1007/s12672-026-04579-z)
Supplement: Supplementary file 1 — Supplementary Material 1 (Figure S1. Validation of candidate gene expression in HNSCC using the GENT2 database. Box plots compare the mRNA expression levels of the eight prognostic genes between Head and neck cancer (red) and Head and neck Normal (blue) tissue samples. Y-axis represents log2-transformed normalized expression values. Seven genes (LAMC2, MFAP2, CTHRC1, CXCL13, FST, SPP1, and PLAU) were significantly overexpressed in tumors (P all<0.01). CDKN2A showed a non-significant trend in this aggregated analysis.). [file 12672_2026_4579_MOESM1_ESM.pdf]

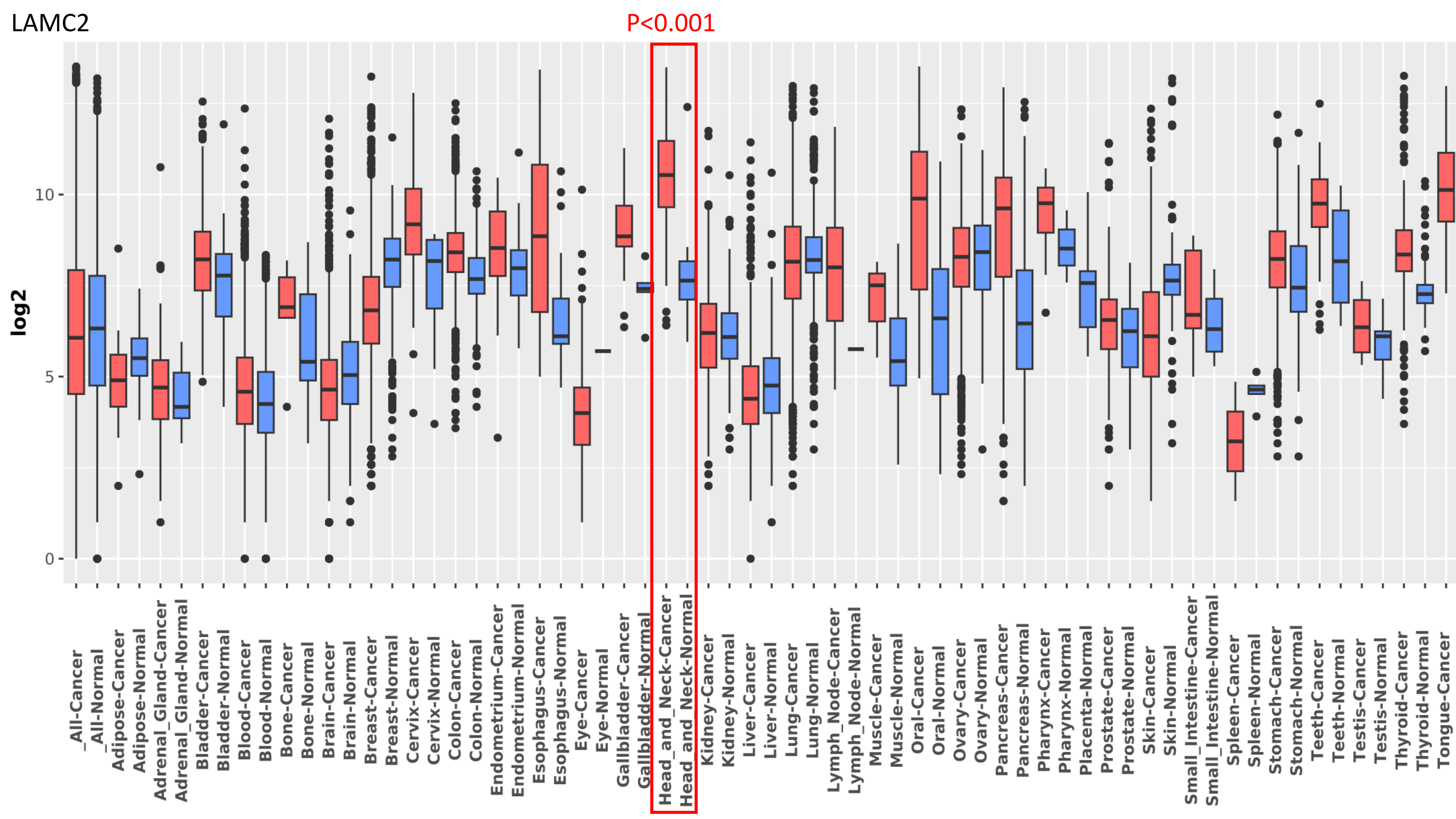
LAMC2
P<0.001
log2
0
5
10
_All-Cancer
_All-Normal
Adipose-Cancer
Adipose-Normal
Adrenal_Gland-Cancer
Adrenal_Gland-Normal
Bladder-Cancer
Bladder-Normal
Blood-Cancer
Blood-Normal
Bone-Cancer
Bone-Normal
Brain-Cancer
Brain-Normal
Breast-Cancer
Breast-Normal
Cervix-Cancer
Cervix-Normal
Colon-Cancer
Colon-Normal
Endometrium-Cancer
Endometrium-Normal
Esophagus-Cancer
Esophagus-Normal
Eye-Cancer
Eye-Normal
Gallbladder-Cancer
Gallbladder-Normal
Head_and_Neck-Cancer
Head_and_Neck-Normal
Kidney-Cancer
Kidney-Normal
Liver-Cancer
Liver-Normal
Lung-Cancer
Lung-Normal
Lymph_Node-Cancer
Lymph_Node-Normal
Muscle-Cancer
Muscle-Normal
Oral-Cancer
Oral-Normal
Ovary-Cancer
Ovary-Normal
Pancreas-Cancer
Pancreas-Normal
Pharynx-Cancer
Pharynx-Normal
Placenta-Normal
Prostate-Cancer
Prostate-Normal
Skin-Cancer
Skin-Normal
Small_Intestine-Cancer
Small_Intestine-Normal
Spleen-Cancer
Spleen-Normal
Stomach-Cancer
Stomach-Normal
Teeth-Cancer
Teeth-Normal
Testis-Cancer
Testis-Normal
Thyroid-Cancer
Thyroid-Normal
Tongue-Cancer

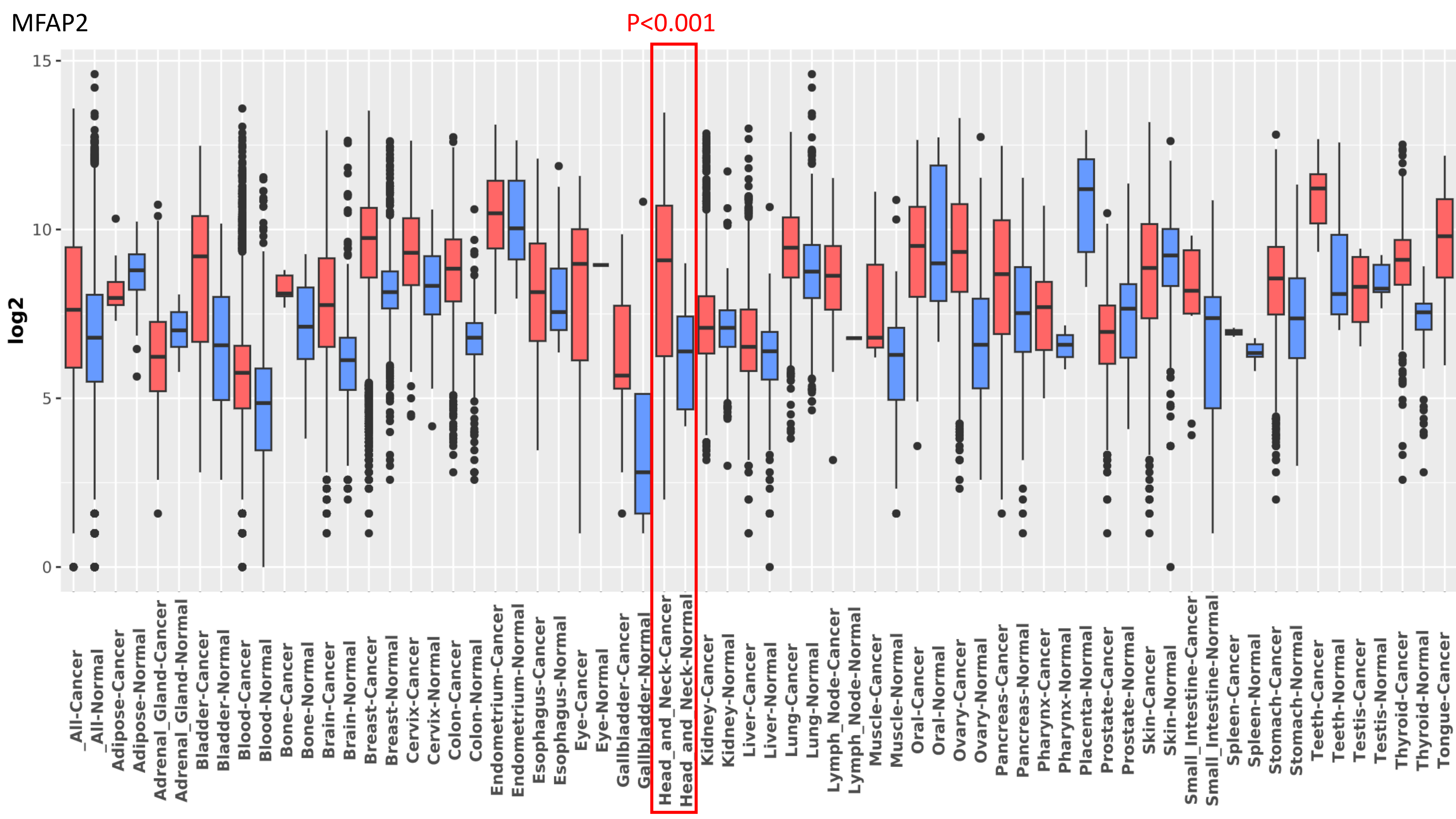
MFAP2
P<0.001
log2
15
10
5
0
_All-Cancer
_All-Normal
Adipose-Cancer
Adipose-Normal
Adrenal_Gland-Cancer
Adrenal_Gland-Normal
Bladder-Cancer
Bladder-Normal
Blood-Cancer
Blood-Normal
Bone-Cancer
Bone-Normal
Brain-Cancer
Brain-Normal
Breast-Cancer
Breast-Normal
Cervix-Cancer
Cervix-Normal
Colon-Cancer
Colon-Normal
Endometrium-Cancer
Endometrium-Normal
Esophagus-Cancer
Esophagus-Normal
Eye-Cancer
Eye-Normal
Gallbladder-Cancer
Gallbladder-Normal
Head_and_Neck-Cancer
Head_and_Neck-Normal
Kidney-Cancer
Kidney-Normal
Liver-Cancer
Liver-Normal
Lung-Cancer
Lung-Normal
Lymph_Node-Cancer
Lymph_Node-Normal
Muscle-Cancer
Muscle-Normal
Oral-Cancer
Oral-Normal
Ovary-Cancer
Ovary-Normal
Pancreas-Cancer
Pancreas-Normal
Pharynx-Cancer
Pharynx-Normal
Placenta-Normal
Prostate-Cancer
Prostate-Normal
Skin-Cancer
Skin-Normal
Small_Intestine-Cancer
Small_Intestine-Normal
Spleen-Cancer
Spleen-Normal
Stomach-Cancer
Stomach-Normal
Teeth-Cancer
Teeth-Normal
Testis-Cancer
Testis-Normal
Thyroid-Cancer
Thyroid-Normal
Tongue-Cancer

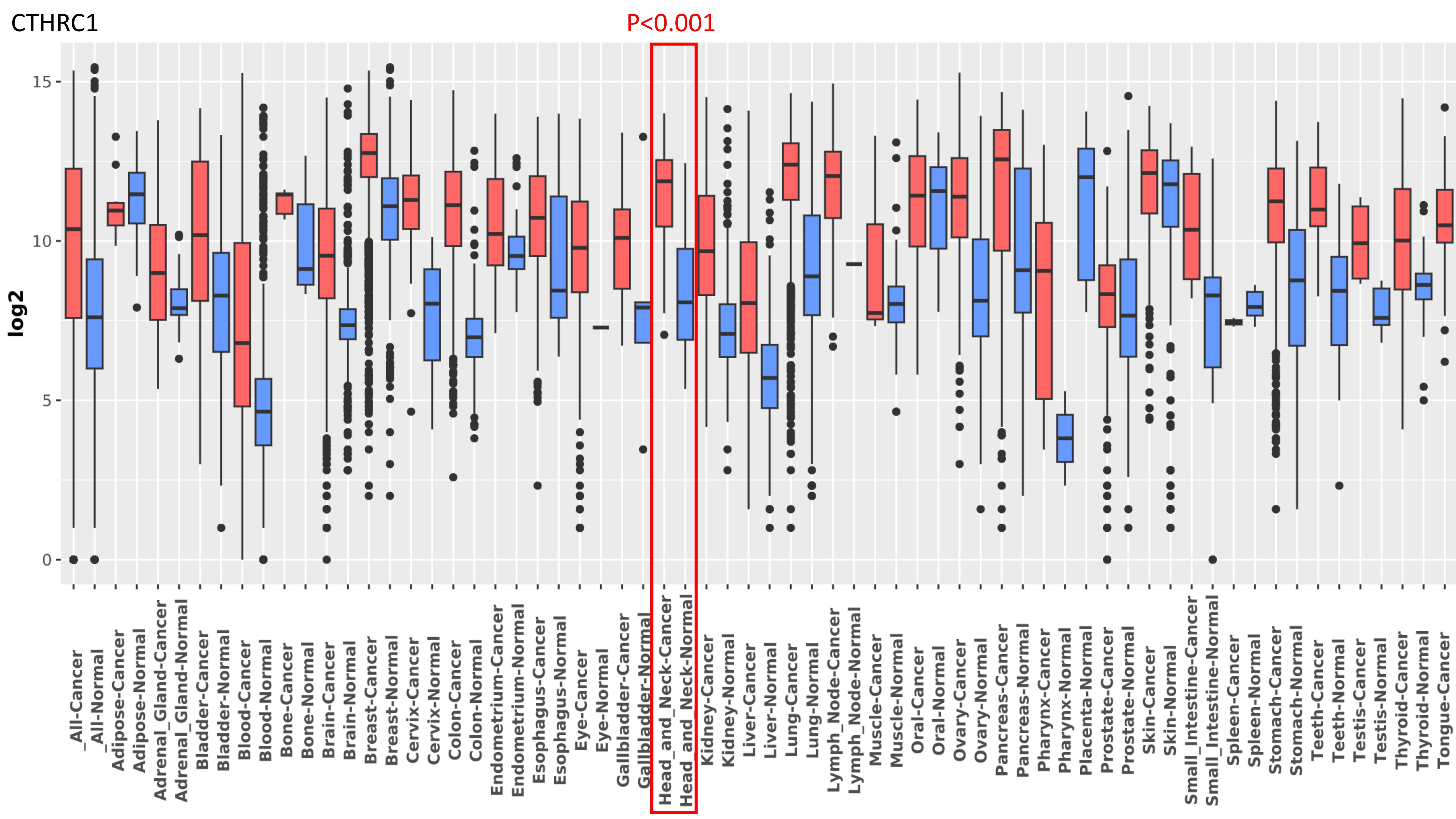

CTHRC1
P<0.001
log2
15
10
5
0
_All-Cancer
_All-Normal
Adipose-Cancer
Adipose-Normal
Adrenal_Gland-Cancer
Adrenal_Gland-Normal
Bladder-Cancer
Bladder-Normal
Blood-Cancer
Blood-Normal
Bone-Cancer
Bone-Normal
Brain-Cancer
Brain-Normal
Breast-Cancer
Breast-Normal
Cervix-Cancer
Cervix-Normal
Colon-Cancer
Colon-Normal
Endometrium-Cancer
Endometrium-Normal
Esophagus-Cancer
Esophagus-Normal
Eye-Cancer
Eye-Normal
Gallbladder-Cancer
Gallbladder-Normal
Head_and_Neck-Cancer
Head_and_Neck-Normal
Kidney-Cancer
Kidney-Normal
Liver-Cancer
Liver-Normal
Lung-Cancer
Lung-Normal
Lymph_Node-Cancer
Lymph_Node-Normal
Muscle-Cancer
Muscle-Normal
Oral-Cancer
Oral-Normal
Ovary-Cancer
Ovary-Normal
Pancreas-Cancer
Pancreas-Normal
Pharynx-Cancer
Pharynx-Normal
Placenta-Normal
Prostate-Cancer
Prostate-Normal
Skin-Cancer
Skin-Normal
Small_Intestine-Cancer
Small_Intestine-Normal
Spleen-Cancer
Spleen-Normal
Stomach-Cancer
Stomach-Normal
Teeth-Cancer
Teeth-Normal
Testis-Cancer
Testis-Normal
Thyroid-Cancer
Thyroid-Normal
Tongue-Cancer

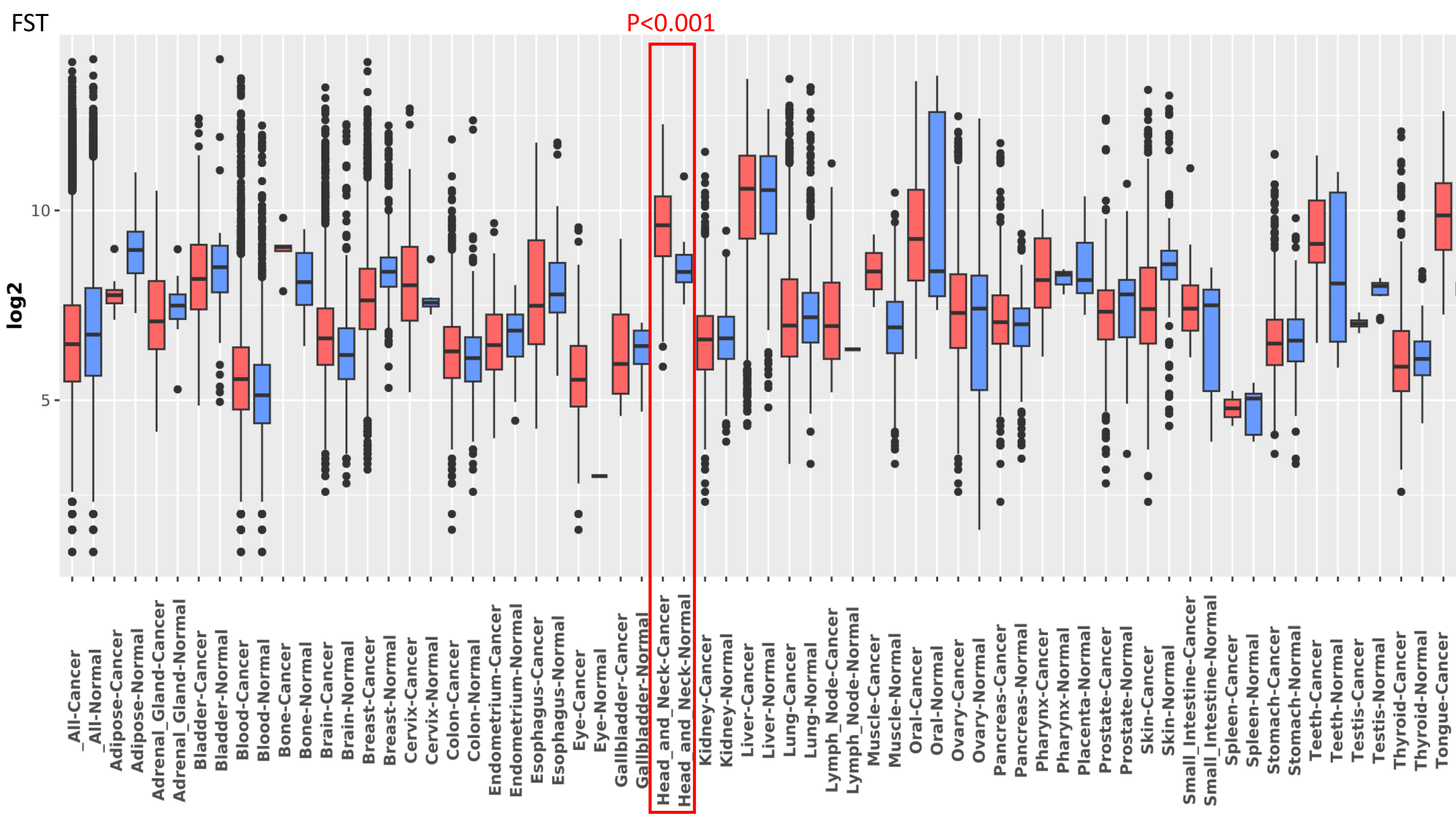

FST
P<0.001
log2
10
5
_All-Cancer
_All-Normal
Adipose-Cancer
Adipose-Normal
Adrenal_Gland-Cancer
Adrenal_Gland-Normal
Bladder-Cancer
Bladder-Normal
Blood-Cancer
Blood-Normal
Bone-Cancer
Bone-Normal
Brain-Cancer
Brain-Normal
Breast-Cancer
Breast-Normal
Cervix-Cancer
Cervix-Normal
Colon-Cancer
Colon-Normal
Endometrium-Cancer
Endometrium-Normal
Esophagus-Cancer
Esophagus-Normal
Eye-Cancer
Eye-Normal
Gallbladder-Cancer
Gallbladder-Normal
Head_and_Neck-Cancer
Head_and_Neck-Normal
Kidney-Cancer
Kidney-Normal
Liver-Cancer
Liver-Normal
Lung-Cancer
Lung-Normal
Lymph_Node-Cancer
Lymph_Node-Normal
Muscle-Cancer
Muscle-Normal
Oral-Cancer
Oral-Normal
Ovary-Cancer
Ovary-Normal
Pancreas-Cancer
Pancreas-Normal
Pharynx-Cancer
Pharynx-Normal
Placenta-Normal
Prostate-Cancer
Prostate-Normal
Skin-Cancer
Skin-Normal
Small_Intestine-Cancer
Small_Intestine-Normal
Spleen-Cancer
Spleen-Normal
Stomach-Cancer
Stomach-Normal
Teeth-Cancer
Teeth-Normal
Testis-Cancer
Testis-Normal
Thyroid-Cancer
Thyroid-Normal
Tongue-Cancer

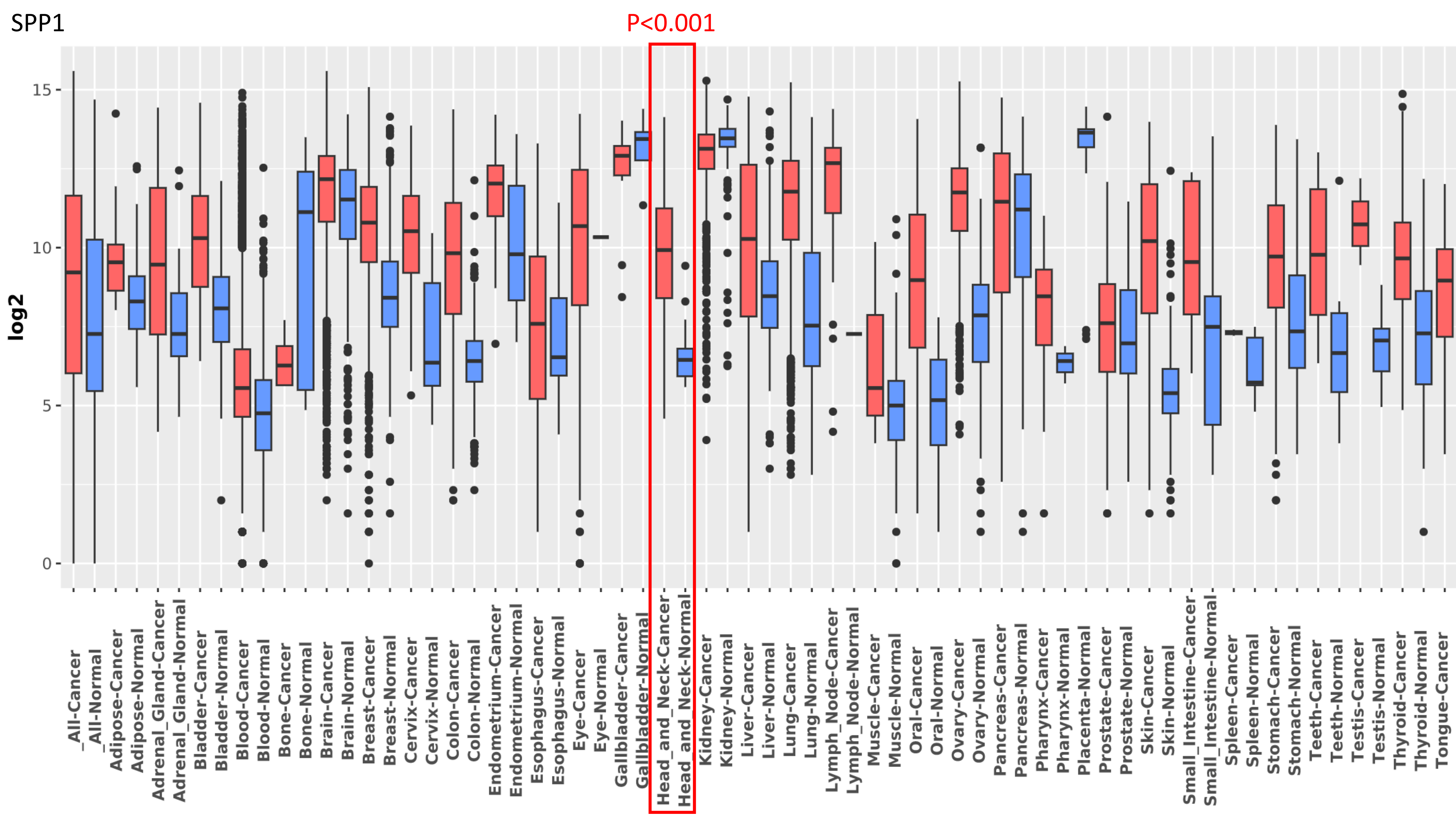
SPP1
P<0.001
log2
15
10
5
0
_All-Cancer
_All-Normal
Adipose-Cancer
Adipose-Normal
Adrenal_Gland-Cancer
Adrenal_Gland-Normal
Bladder-Cancer
Bladder-Normal
Blood-Cancer
Blood-Normal
Bone-Cancer
Bone-Normal
Brain-Cancer
Brain-Normal
Breast-Cancer
Breast-Normal
Cervix-Cancer
Cervix-Normal
Colon-Cancer
Colon-Normal
Endometrium-Cancer
Endometrium-Normal
Esophagus-Cancer
Esophagus-Normal
Eye-Cancer
Eye-Normal
Gallbladder-Cancer
Gallbladder-Normal
Head_and_Neck-Cancer
Head_and_Neck-Normal
Kidney-Cancer
Kidney-Normal
Liver-Cancer
Liver-Normal
Lung-Cancer
Lung-Normal
Lymph_Node-Cancer
Lymph_Node-Normal
Muscle-Cancer
Muscle-Normal
Oral-Cancer
Oral-Normal
Ovary-Cancer
Ovary-Normal
Pancreas-Cancer
Pancreas-Normal
Pharynx-Cancer
Pharynx-Normal
Placenta-Normal
Prostate-Cancer
Prostate-Normal
Skin-Cancer
Skin-Normal
Small_Intestine-Cancer
Small_Intestine-Normal
Spleen-Cancer
Spleen-Normal
Stomach-Cancer
Stomach-Normal
Teeth-Cancer
Teeth-Normal
Testis-Cancer
Testis-Normal
Thyroid-Cancer
Thyroid-Normal
Tongue-Cancer

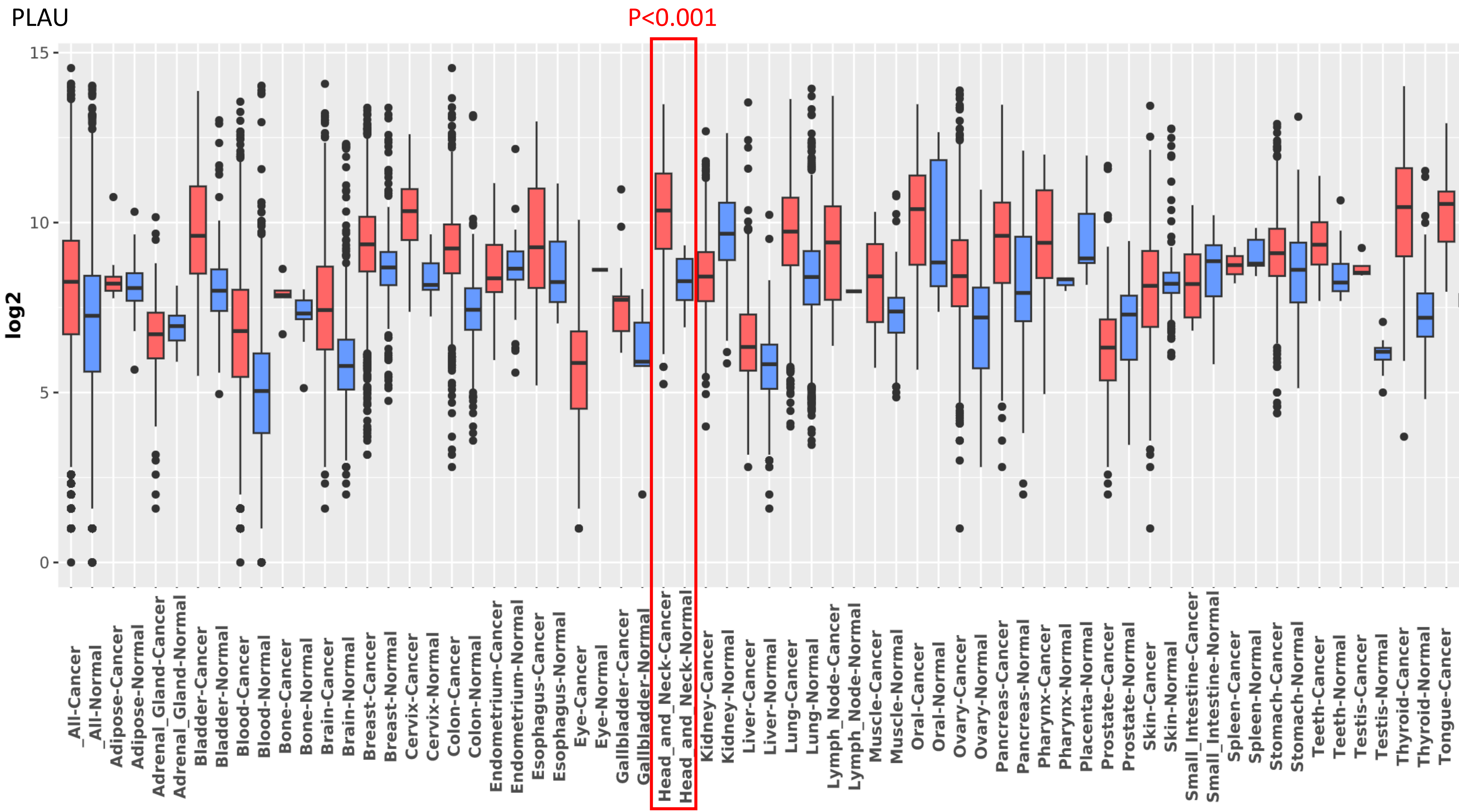

PLAU
P<0.001
log2
15
10
5
0
_All-Cancer
_All-Normal
Adipose-Cancer
Adipose-Normal
Adrenal_Gland-Cancer
Adrenal_Gland-Normal
Bladder-Cancer
Bladder-Normal
Blood-Cancer
Blood-Normal
Bone-Cancer
Bone-Normal
Brain-Cancer
Brain-Normal
Breast-Cancer
Breast-Normal
Cervix-Cancer
Cervix-Normal
Colon-Cancer
Colon-Normal
Endometrium-Cancer
Endometrium-Normal
Esophagus-Cancer
Esophagus-Normal
Eye-Cancer
Eye-Normal
Gallbladder-Cancer
Gallbladder-Normal
Head_and_Neck-Cancer
Head_and_Neck-Normal
Kidney-Cancer
Kidney-Normal
Liver-Cancer
Liver-Normal
Lung-Cancer
Lung-Normal
Lymph_Node-Cancer
Lymph_Node-Normal
Muscle-Cancer
Muscle-Normal
Oral-Cancer
Oral-Normal
Ovary-Cancer
Ovary-Normal
Pancreas-Cancer
Pancreas-Normal
Pharynx-Cancer
Pharynx-Normal
Placenta-Normal
Prostate-Cancer
Prostate-Normal
Skin-Cancer
Skin-Normal
Small_Intestine-Cancer
Small_Intestine-Normal
Spleen-Cancer
Spleen-Normal
Stomach-Cancer
Stomach-Normal
Teeth-Cancer
Teeth-Normal
Testis-Cancer
Testis-Normal
Thyroid-Cancer
Thyroid-Normal
Tongue-Cancer

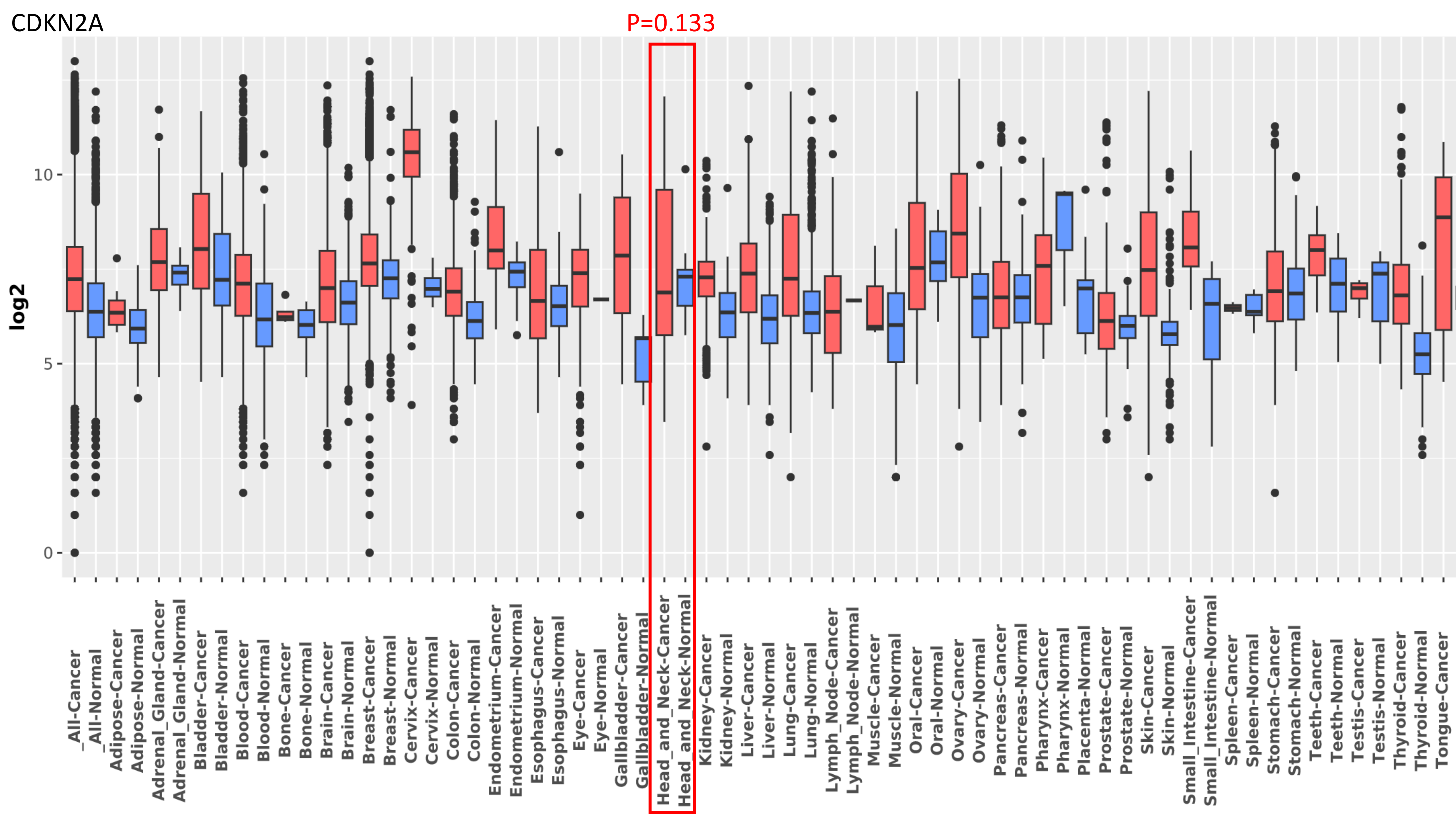

CDKN2A
P=0.133
log2
10
5
0
_All-Cancer
_All-Normal
Adipose-Cancer
Adipose-Normal
Adrenal_Gland-Cancer
Adrenal_Gland-Normal
Bladder-Cancer
Bladder-Normal
Blood-Cancer
Blood-Normal
Bone-Cancer
Bone-Normal
Brain-Cancer
Brain-Normal
Breast-Cancer
Breast-Normal
Cervix-Cancer
Cervix-Normal
Colon-Cancer
Colon-Normal
Endometrium-Cancer
Endometrium-Normal
Esophagus-Cancer
Esophagus-Normal
Eye-Cancer
Eye-Normal
Gallbladder-Cancer
Gallbladder-Normal
Head_and_Neck-Cancer
Head_and_Neck-Normal
Kidney-Cancer
Kidney-Normal
Liver-Cancer
Liver-Normal
Lung-Cancer
Lung-Normal
Lymph_Node-Cancer
Lymph_Node-Normal
Muscle-Cancer
Muscle-Normal
Oral-Cancer
Oral-Normal
Ovary-Cancer
Ovary-Normal
Pancreas-Cancer
Pancreas-Normal
Pharynx-Cancer
Pharynx-Normal
Placenta-Normal
Prostate-Cancer
Prostate-Normal
Skin-Cancer
Skin-Normal
Small_Intestine-Cancer
Small_Intestine-Normal
Spleen-Cancer
Spleen-Normal
Stomach-Cancer
Stomach-Normal
Teeth-Cancer
Teeth-Normal
Testis-Cancer
Testis-Normal
Thyroid-Cancer
Thyroid-Normal
Tongue-Cancer

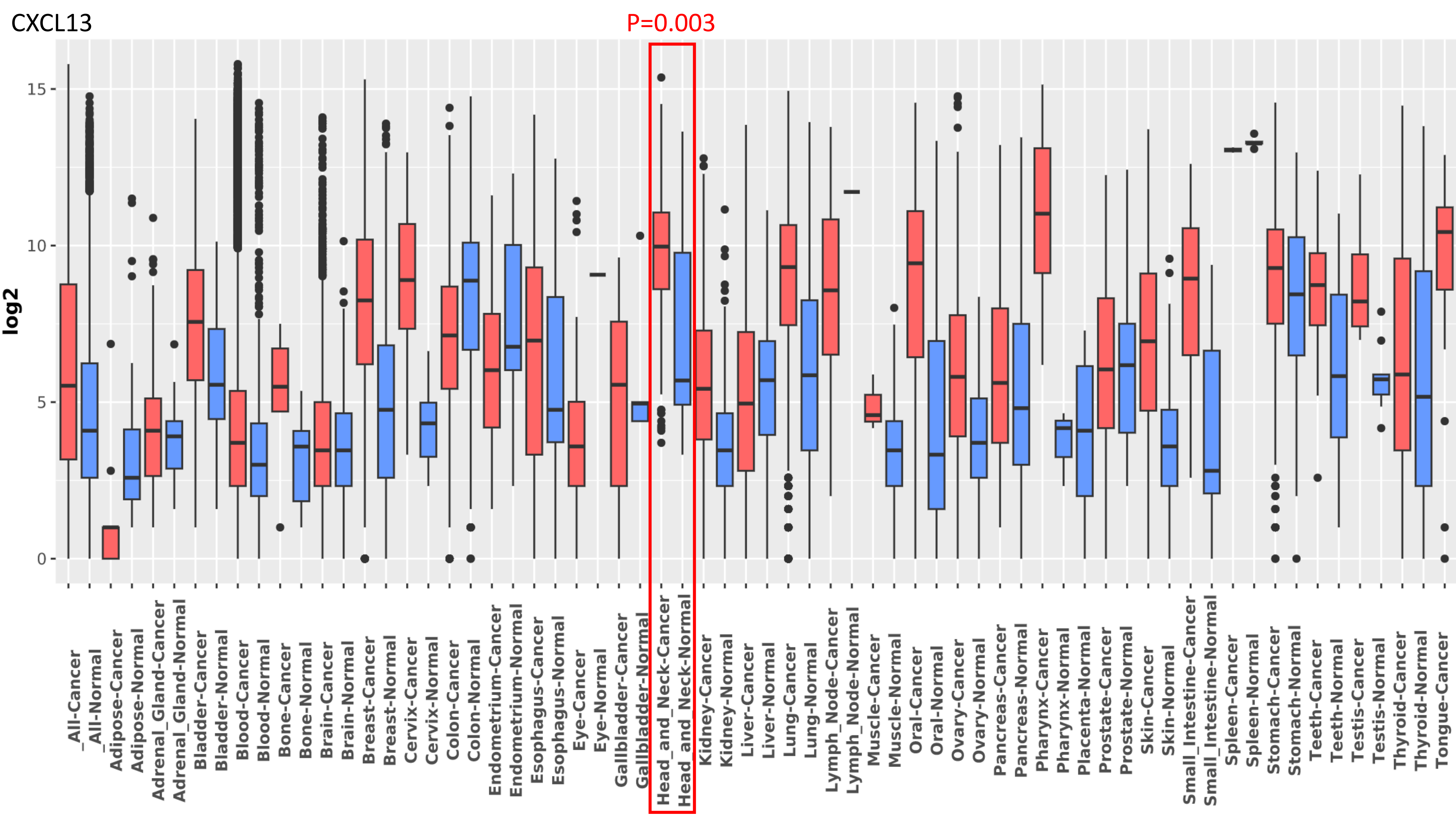

CXCL13
P=0.003
log2
15
10
5
0
_All-Cancer
_All-Normal
Adipose-Cancer
Adipose-Normal
Adrenal_Gland-Cancer
Adrenal_Gland-Normal
Bladder-Cancer
Bladder-Normal
Blood-Cancer
Blood-Normal
Bone-Cancer
Bone-Normal
Brain-Cancer
Brain-Normal
Breast-Cancer
Breast-Normal
Cervix-Cancer
Cervix-Normal
Colon-Cancer
Colon-Normal
Endometrium-Cancer
Endometrium-Normal
Esophagus-Cancer
Esophagus-Normal
Eye-Cancer
Eye-Normal
Gallbladder-Cancer
Gallbladder-Normal
Head_and_Neck-Cancer
Head_and_Neck-Normal
Kidney-Cancer
Kidney-Normal
Liver-Cancer
Liver-Normal
Lung-Cancer
Lung-Normal
Lymph_Node-Cancer
Lymph_Node-Normal
Muscle-Cancer
Muscle-Normal
Oral-Cancer
Oral-Normal
Ovary-Cancer
Ovary-Normal
Pancreas-Cancer
Pancreas-Normal
Pharynx-Cancer
Pharynx-Normal
Placenta-Normal
Prostate-Cancer
Prostate-Normal
Skin-Cancer
Skin-Normal
Small_Intestine-Cancer
Small_Intestine-Normal
Spleen-Cancer
Spleen-Normal
Stomach-Cancer
Stomach-Normal
Teeth-Cancer
Teeth-Normal
Testis-Cancer
Testis-Normal
Thyroid-Cancer
Thyroid-Normal
Tongue-Cancer
