# Supplementary figures and images for: HPV status determines prognostic gene expression methylation and immune infiltration in head and neck squamous cell carcinoma
Source: Discov Oncol. 2026 Mar 3;17:553. doi: 10.1007/s12672-026-04579-z (PMC13065962; doi:10.1007/s12672-026-04579-z)

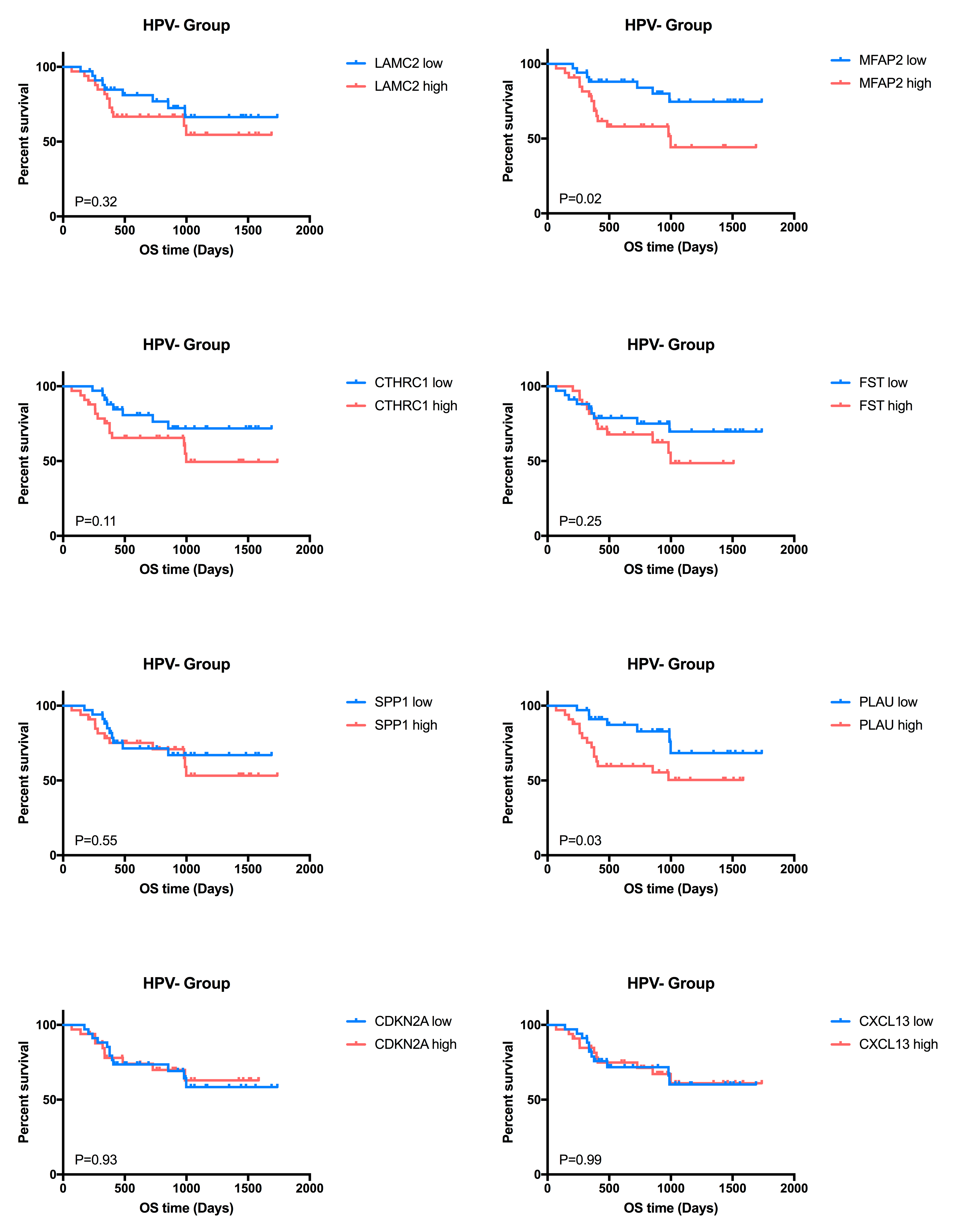

Supplement: Supplementary file 2 — Supplementary Material 2 (Figure S2. HPV-stratified survival analysis of candidate genes in the TCGA-HNSC cohort. Kaplan-Meier curves depict overall survival in the HPV- subgroup stratified by high vs. low expression (median cut-off) of LAMC2, MFAP2, CTHRC1, FST, SPP1, PLAU, CDKN2A, and CXCL13. Log-rank p-values are indicated. Analysis of the HPV+ subgroup was not feasible due to an insufficient number of survival events.). [file 12672_2026_4579_MOESM2_ESM.tiff]
