# Supplementary material for: HPV status determines prognostic gene expression methylation and immune infiltration in head and neck squamous cell carcinoma
Source: Discov Oncol. 2026 Mar 3;17:553. doi: 10.1007/s12672-026-04579-z (PMC13065962; doi:10.1007/s12672-026-04579-z)
Supplement: Supplementary file 3 — Supplementary Material 3 (Figure S3. Multi-region methylation analysis of eight prognostic genes in HPV- versus HPV+ HNSCC. Comparative methylation profiles across genomic features. Bar graphs display the methylation β-values for representative probes of each gene in HPV- tumors versus HPV+ tumors. Probes are grouped by genomic feature: Promoter, Gene Body, and CpG islands. Only features with available probe data are shown.). [file 12672_2026_4579_MOESM3_ESM.pdf]

LAMC2

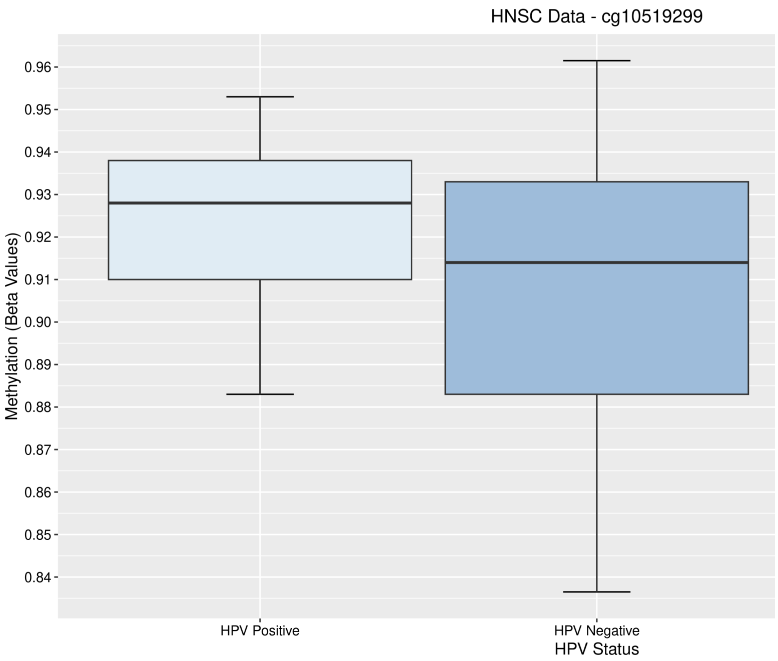

Promoter
P= 3.32e-7

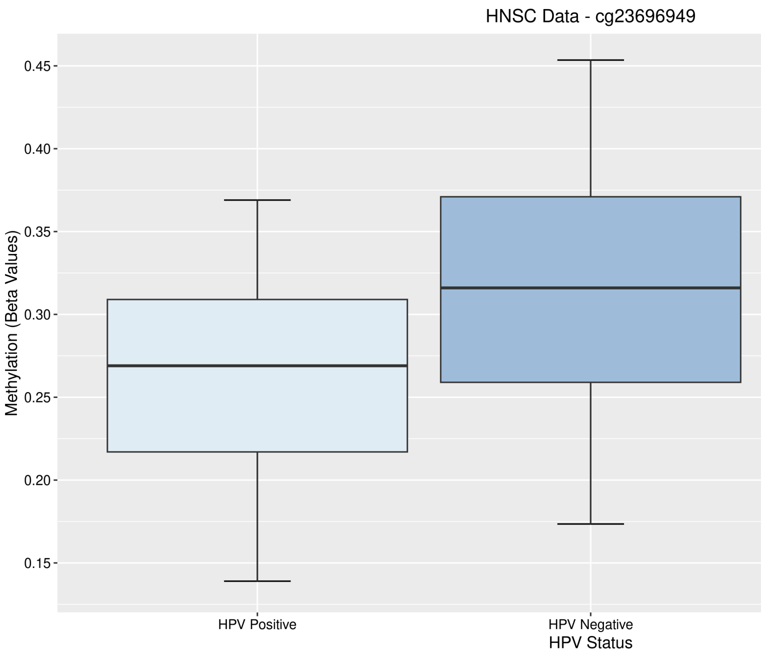

Gene body
P=2.9e-8

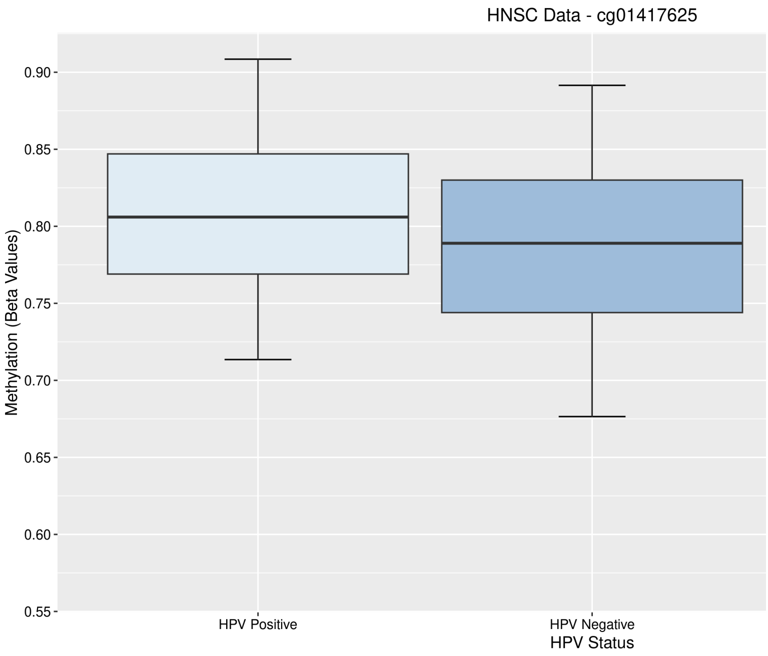

Gene body
P=2.8e-3

MFAP2

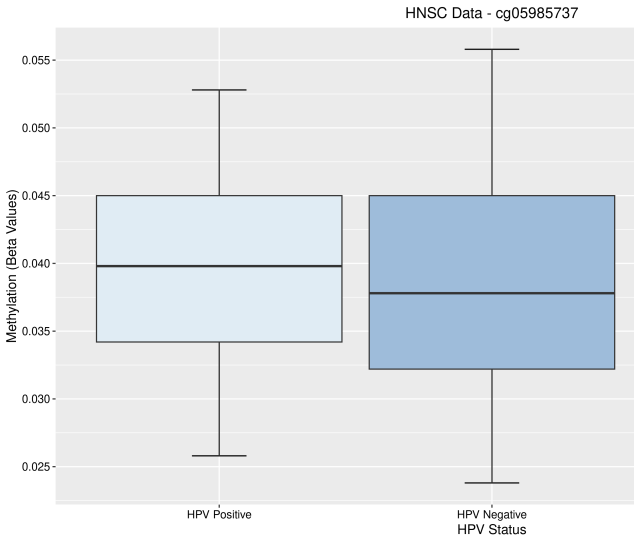

Promoter
P= 6.16e-16

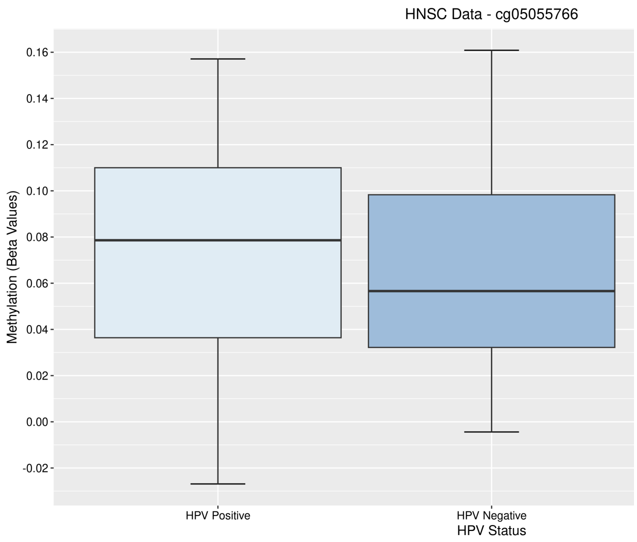

Gene body
P= 1.6e-2

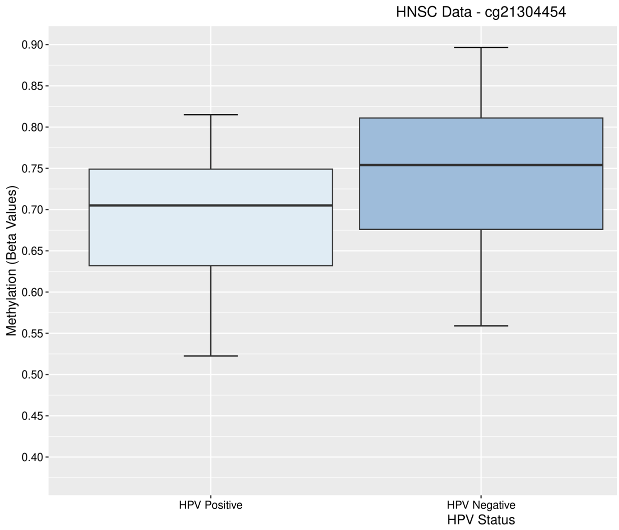

Gene body
P= 4.25e-6

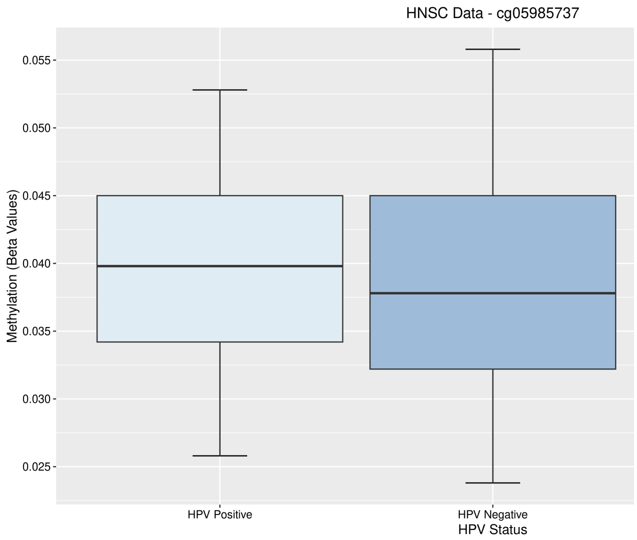

CpG Island
P= 6.16e-16

CTHRC1

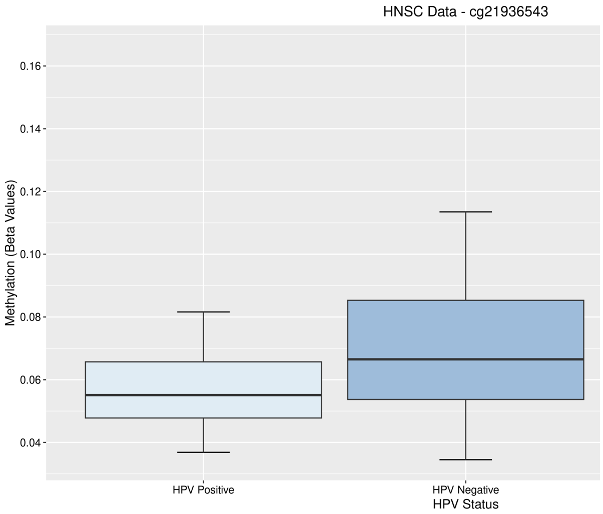

Promoter
P= 8.13e-13

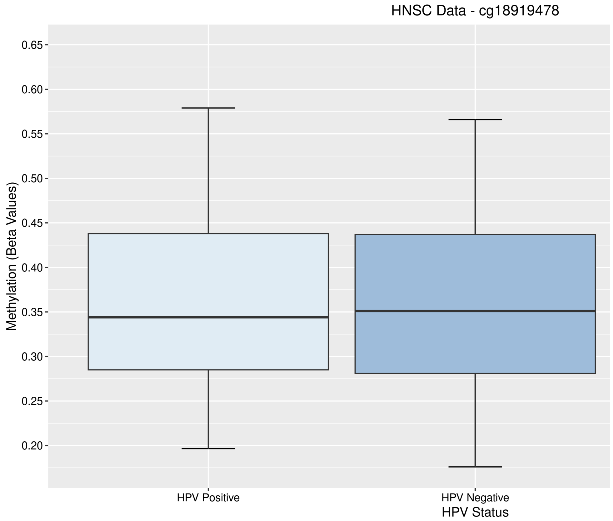

Gene body
P= 9.02e-3

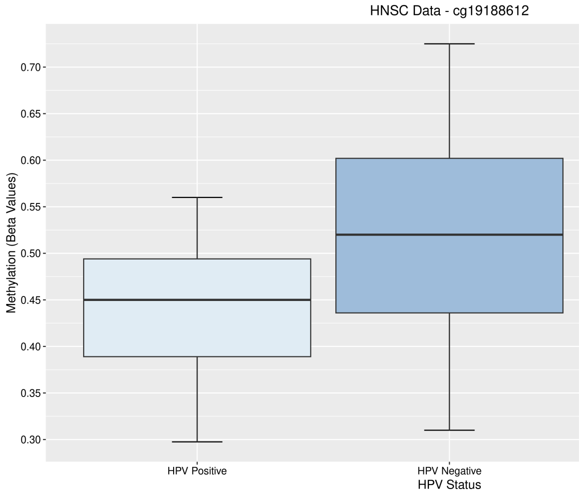

Gene body
P= 9.93e-3

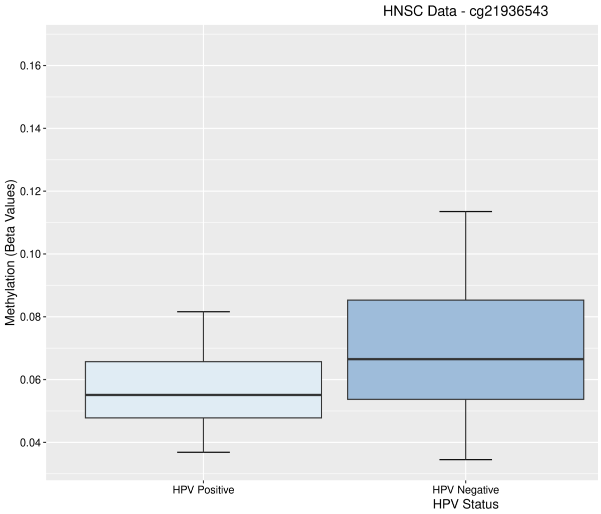

CpG Island
P= 8.13e-13

FST

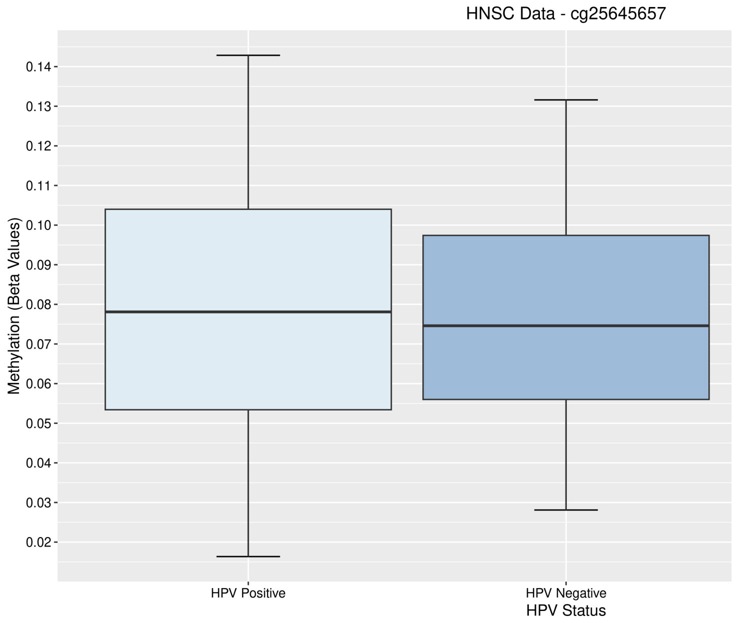

Promoter
P= 6.84e-11

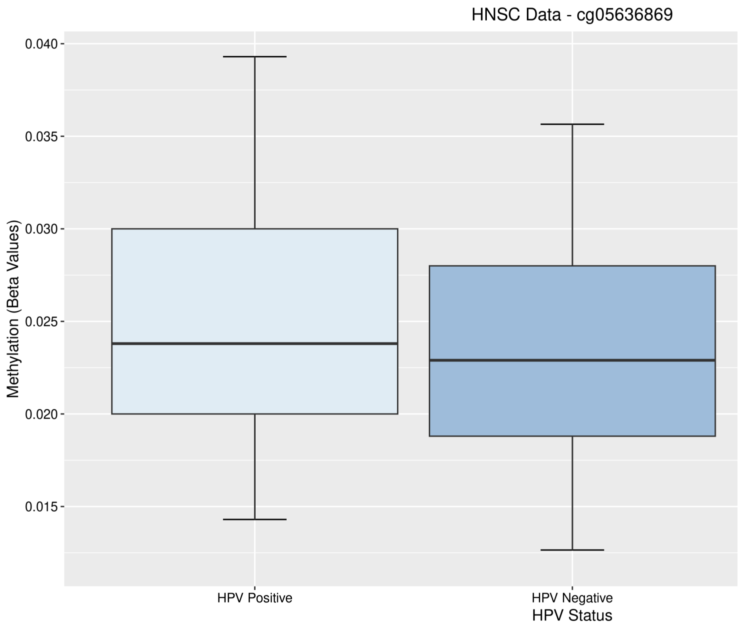

Gene body
P= 1.33e-2

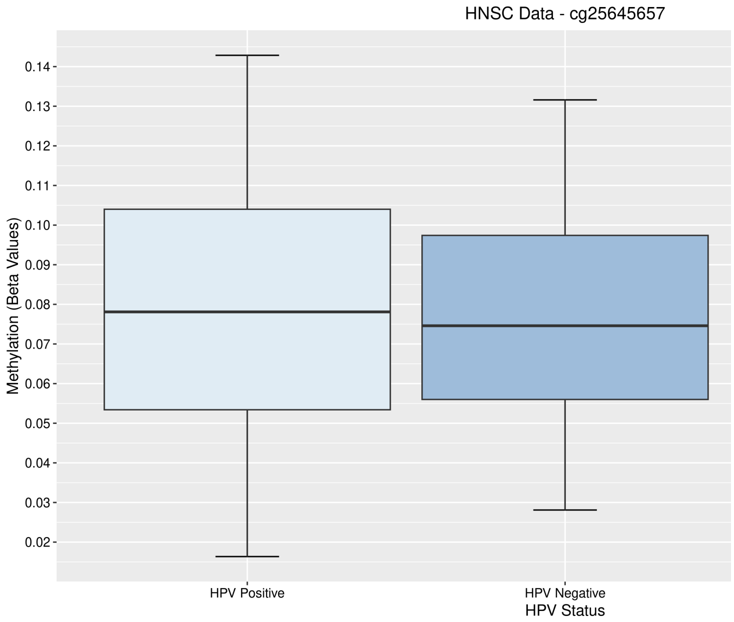

CpG Island
P= 6.84e-11

SPP1

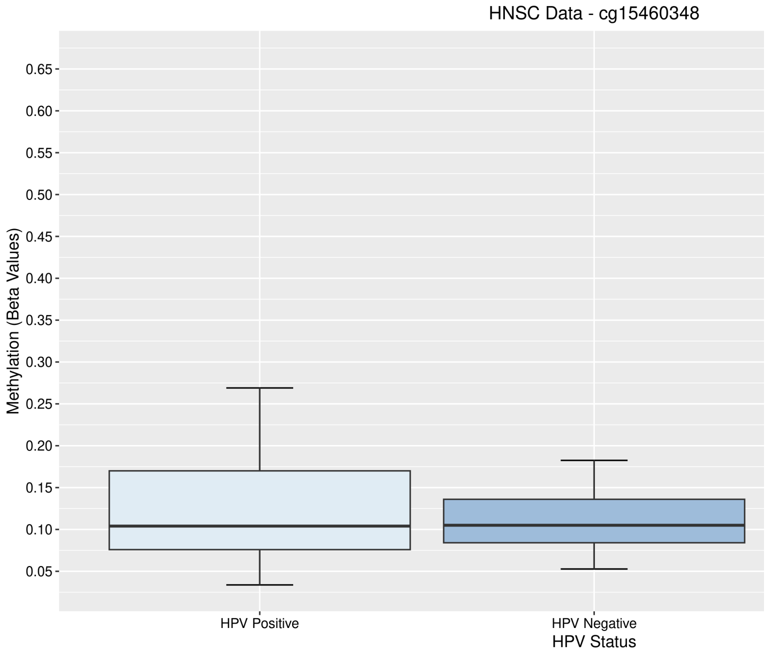

Promoter
P= 1.94e-7

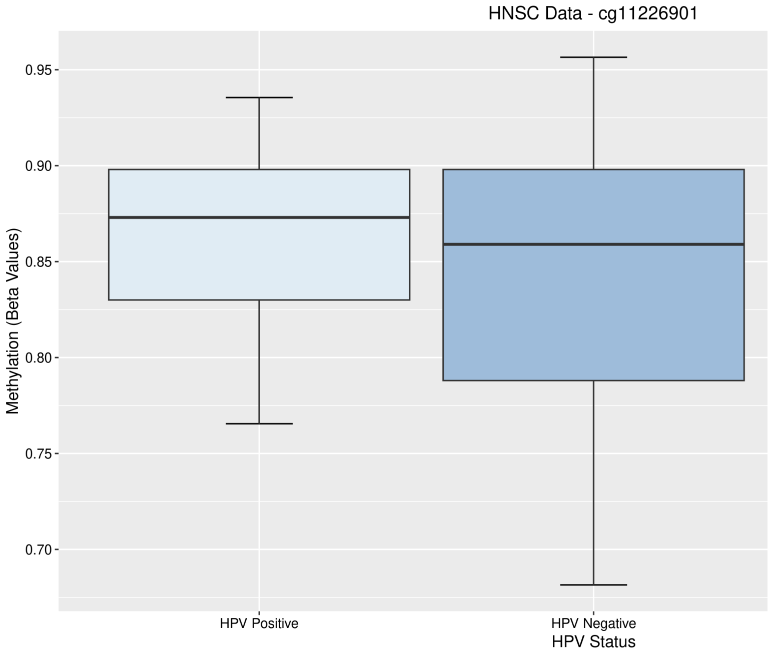

Gene body
P= 1.6e-7

PLAU

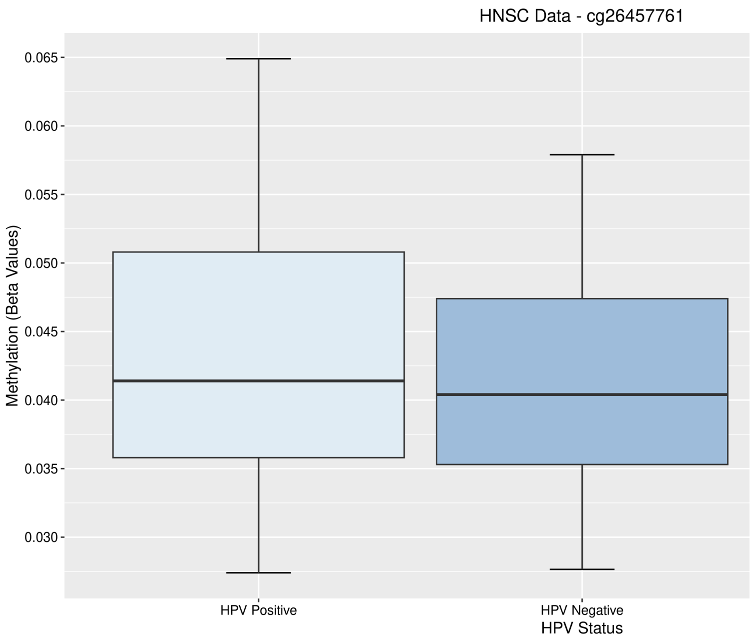

Promoter
P= 1.35e-17

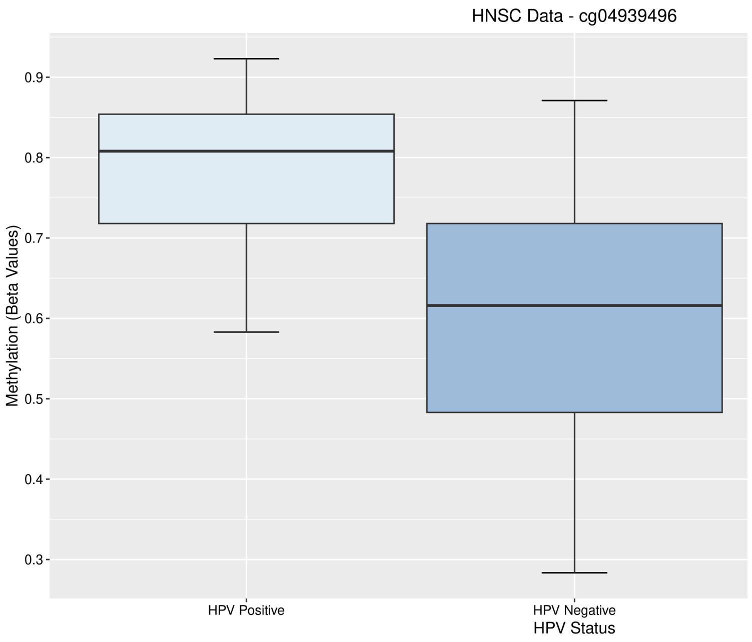

Gene body
P= 2.04e-13

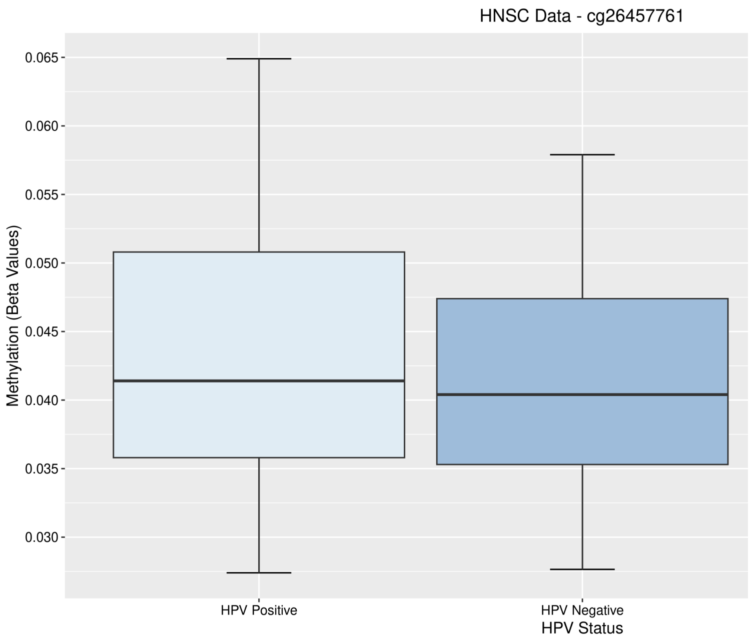

CpG Island
P= 1.35e-17

CDKN2A

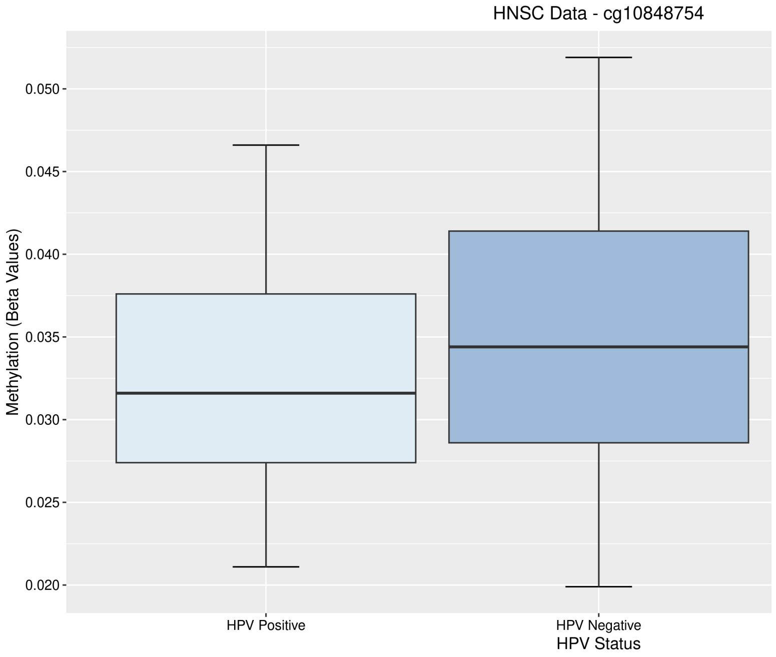

Promoter
P= 4.02e-3

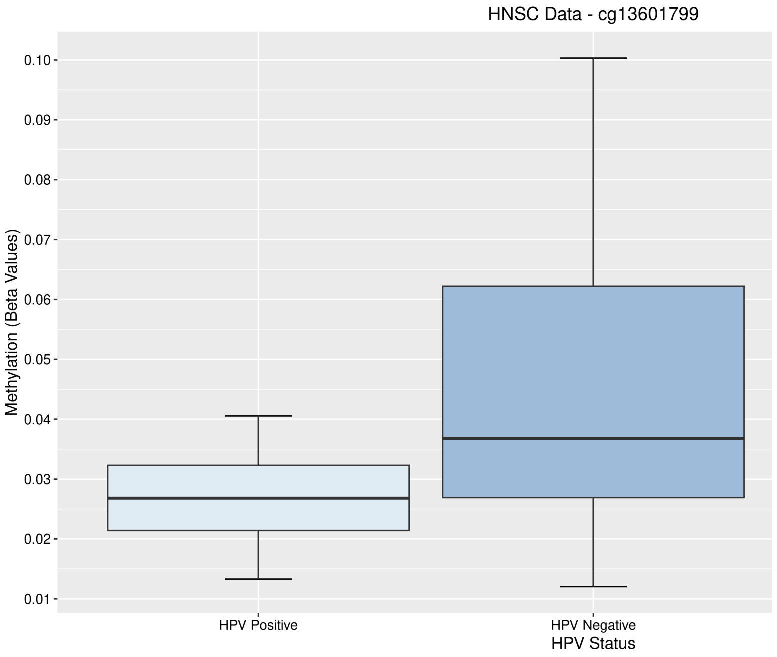

Gene body
P= 6.08e-10

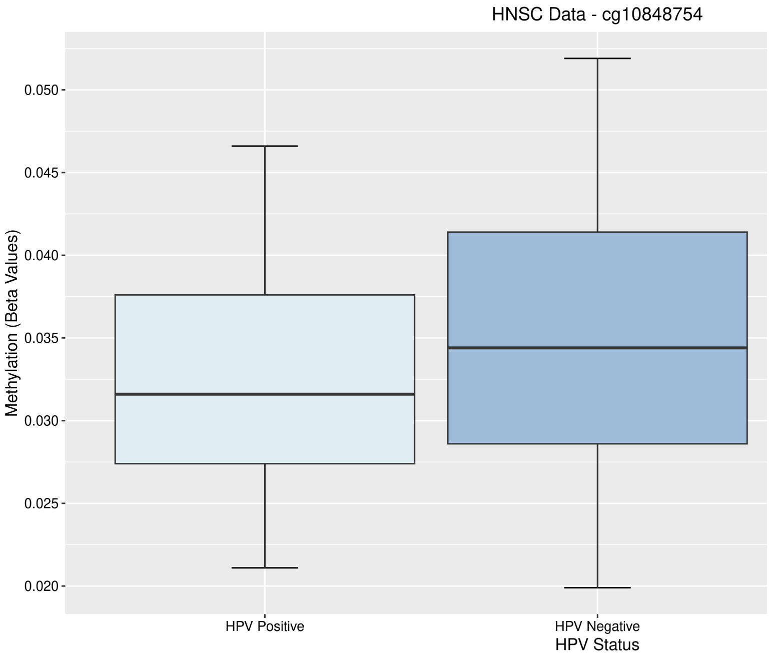

CpG Island
P= 4.02e-3

CXCL13

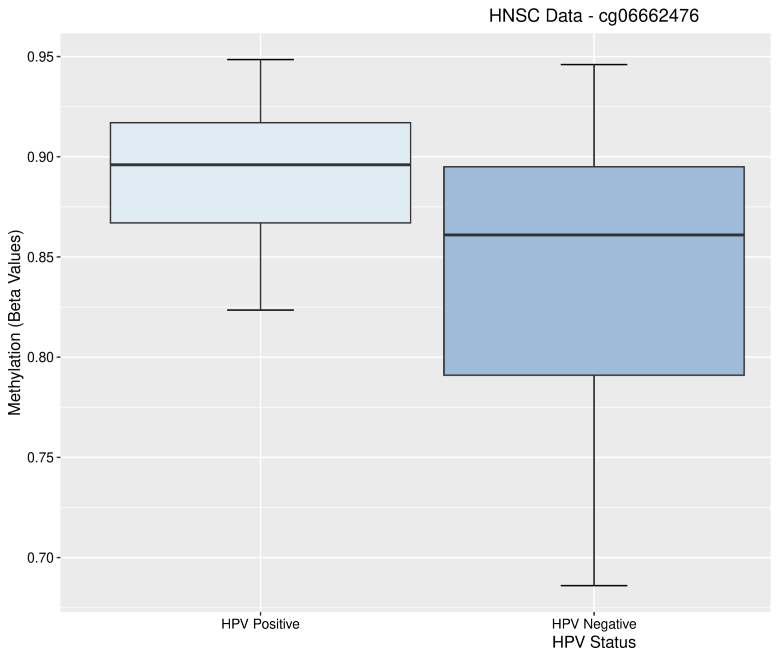

Gene body
P= 2.08e-4
